# Network-specific sex differentiation of intrinsic brain function in males with autism

**DOI:** 10.1186/s13229-018-0192-x

**Published:** 2018-03-06

**Authors:** Dorothea L. Floris, Meng-Chuan Lai, Tanmay Nath, Michael P. Milham, Adriana Di Martino

**Affiliations:** 1 Hassenfeld Children’s Hospital at NYU Langone Health, Department of Child and Adolescent Psychiatry, Child Study Center, 1 Park Avenue, New York City, NY 10016 USA; 2 Child and Youth Mental Health Collaborative at the Centre for Addiction and Mental Health and The Hospital for Sick Children, Department of Psychiatry, University of Toronto, Toronto, ON M6J 1H4 Canada; 3 Autism Research Centre, Department of Psychiatry, University of Cambridge, Cambridge, CB2 8AH UK; 4 Center for the Developing Brain, Child Mind Institute, New York, NY 10022 USA; 5 Nathan S Kline Institute for Psychiatric Research, Orangeburg, NY 10962 USA

**Keywords:** Autism spectrum disorder, Resting-state fMRI, Sex differentiation, Sex mosaicism, Extreme Male Brain, Gender Incoherence

## Abstract

**Background:**

The male predominance in the prevalence of autism spectrum disorder (ASD) has motivated research on sex differentiation in ASD. Multiple sources of evidence have suggested a neurophenotypic convergence of ASD-related characteristics and typical sex differences. Two existing, albeit competing, models provide predictions on such neurophenotypic convergence. These two models are testable with neuroimaging. Specifically, the Extreme Male Brain (EMB) model predicts that ASD is associated with enhanced brain maleness in both males and females with ASD (i.e., a shift-towards-maleness). In contrast, the Gender Incoherence (GI) model predicts a shift-towards-maleness in females, yet a shift-towards-femaleness in males with ASD.

**Methods:**

To clarify whether either model applies to the intrinsic functional properties of the brain in males with ASD, we measured the statistical overlap between typical sex differences and ASD-related atypicalities in resting-state fMRI (R-fMRI) datasets largely available in males. Main analyses focused on two large-scale R-fMRI samples: 357 neurotypical (NT) males and 471 NT females from the 1000 Functional Connectome Project and 360 males with ASD and 403 NT males from the Autism Brain Imaging Data Exchange.

**Results:**

Across all R-fMRI metrics, results revealed coexisting, but network-specific, shift-towards-maleness and shift-towards-femaleness in males with ASD. A shift-towards-maleness mostly involved the default network, while a shift-towards-femaleness mostly occurred in the somatomotor network. Explorations of the associated cognitive processes using available cognitive ontology maps indicated that higher-order social cognitive functions corresponded to the shift-towards-maleness, while lower-order sensory motor processes corresponded to the shift-towards-femaleness.

**Conclusions:**

The present findings suggest that atypical intrinsic brain properties in males with ASD partly reflect mechanisms involved in sexual differentiation. A model based on network-dependent atypical sex mosaicism can synthesize prior competing theories on factors involved in sex differentiation in ASD.

**Electronic supplementary material:**

The online version of this article (10.1186/s13229-018-0192-x) contains supplementary material, which is available to authorized users.

## Background

Substantial evidence supports models of autism spectrum disorder (ASD) as a condition characterized by altered brain connectivity [1, 2]. Yet, the striking heterogeneity of ASD has challenged efforts to profile the mechanisms underlying ASD-related dysconnections. One significant source of heterogeneity in ASD is biological sex [3, 4], as highlighted by the 3–4:1 male preponderance in prevalence [5, 6]. However, the impact of biological sex on ASD-related dysonnections remains poorly understood. In recent years, resting-state functional magnetic resonance imaging (R-fMRI) has demonstrated its feasibility in capturing typical sex differences in various aspects of the intrinsic connectome [7–9] and has emerged as a robust tool for substantiating the functional dysconnectivity hypothesis of ASD [1, 2]. This has motivated initial R-fMRI studies to characterize the contribution of biological sex to the neurobiology of ASD [10, 11]. Preliminary evidence suggests that both sex-dependent and sex-independent dysconnections coexist in ASD [12].

Here, in considering sex-dependent factors, we focused on investigating the neurophenotypic convergence of ASD-related characteristics and typical sex differences [13, 14]. Our focus was motivated by (1) genetic evidence emphasizing shared mechanisms between vulnerability to ASD and typical sexual differentiation [15] and (2) two current theoretical models whose predictions on neurophenotypic convergence can be assessed with neuroimaging. One model is the Extreme Male Brain (EMB) theory, which emerges from observations at the cognitive-behavioral level that individuals with ASD, regardless of sex, show “masculinized” or “hypermasculinized” profiles of empathizing and systemizing [16, 17]. Neurophenotypic prediction from the EMB model is that ASD brain characteristics will be associated with enhanced brain maleness (i.e., *shift-towards-maleness*) in both males and females with ASD. The other model is the Gender Incoherence (GI; [18]) theory. This is grounded on anthropometric and physiological findings that adult males and females with ASD are more androgynous than same-sex neurotypical peers [18]. The neurophenotypic prediction of the GI model is that a *shift-towards-maleness* is limited to females with ASD and that males with ASD instead show a *shift-towards-femaleness*.

While neuroimaging studies have begun to consider the mechanisms underlying sex differentiation in ASD in the context of these two models [14], only two studies have focused on intrinsic functional properties of the connectome. Both R-fMRI studies converged on a neural shift-towards-maleness in females with ASD, consistent with a prior volumetric study [3]. However, these earlier studies diverged in regard to males with ASD. Ypma et al. [11] reported a shift-towards-maleness within the default network (DN) in individuals with ASD regardless of their biological sex—a pattern consistent with EMB predictions. Results showed that both males and females with ASD exhibited hypo-connectivity within DN compared to typical males, who, in turn, showed lower intrinsic functional connectivity (iFC) than typical females. Alaerts et al. [10] found a similar pattern of a shift-towards-maleness in females with ASD in the voxel-wise iFC of the posterior superior temporal sulcus and posterior cingulate cortex (PCC). However, they also observed a shift-towards-femaleness in males with ASD—as predicted by GI. The disparities in these R-fMRI studies may reflect their moderate sample sizes. They employed a two-factorial design (i.e., testing sex-by-diagnosis interactions), with sample size generally including *n* ~ 40 individuals per cell. This is due to the scarcity of available ASD female datasets even in large repositories such as the Autism Brain Imaging Data Exchange (ABIDE).

Conjunction (spatial overlap) analyses can be a useful alternative and complementary approach in the identification of potential associations between ASD-related atypicalities and typical sex difference maps [3, 14]. This is particularly true in instances of limited availability of female datasets. Factorial designs can provide information about factors related to typical sex differentiation in regions that *differ* between males and females with ASD relative to NT males and females (i.e., by testing for regions showing significant sex-by-diagnosis interactions) and brain regions showing main effects of sex (ignoring diagnostic group) or ASD (ignoring sex). However, it is still possible that regions showing ASD-related atypicalities do not differ between males and females with ASD relative to NT males and females but are still related to typical sex differentiation. These nuances can be captured in the examination of specific spatial overlap scenarios of effects predicted by hypothetical links between ASD atypicalities (separately for ASD males and ASD females, whenever data is available) and typical sex differences.

We applied conjunction analyses to the large ABIDE I male R-fMRI dataset, thus allowing to address concerns on sample size as well as prior inconsistencies in sex-dependent intrinsic functional brain properties in males with ASD [10, 11]. We focused on five key whole-brain voxel-wise R-fMRI indices in regard to (a) typical sex differences in a neurotypical (NT) sample from the 1000 Functional Connectome Project (FCP) [19] and (b) ASD-related differences among males from ABIDE I [1]. We conducted an unbiased whole-brain exploration given reports of multiple networks involved in ASD including and beyond DN. To ensure that findings were not dependent on the specific NT sample employed, we examined replicability using the Brain Genomics Superstruct Project (GSP) [20] sample.

## Methods

### Analytical overview

Using conjunction analyses, we explored whether atypical intrinsic brain properties in males with ASD showed significant spatial overlap with those exhibiting typical sex differences. Primary analyses used group difference *Z*-statistics maps from two published large-scale R-fMRI studies [1, 19]. Applying a more stringent statistical voxel-level threshold of *Z* ≥ 2.58 (cluster level *p* < 0.05) yielded highly similar findings as original results; see Additional file 1: Figure S1 for illustrative purposes. These studies were selected for their large sample sizes and for the representativeness of their findings in regard to typical sex differentiation (e.g., [7–9, 21, 22]) as well as ASD-related atypicalities [2, 23–25] (for complete reviews, see [26]). Five R-fMRI metrics were examined which have been reported to reflect typical sex differences [7–9, 19] and ASD-related intrinsic brain properties [1, 24, 27, 28]: (1) regional homogeneity (ReHo) [29], (2) voxel-mirrored homotopic connectivity (VMHC) [30], (3) network degree centrality (DC) [31], (4) PCC-iFC, and (5) fractional amplitude of low frequency fluctuations (fALFF) [32] (for details on each measure, see Additional file 2: Supplementary methods). Validation and replication analytical strategies were conducted to address the possible confounds related to samples and preprocessing differences (Table 1).

**Table 1:** Overview of analysis strategies

Strategy	Description of overlapping maps	Addressed potential confounds	Samples
Strategy 1 *Originally published Z-maps*	*Z*-maps obtained from original ABIDE I and FCP samples	–	ABIDE I: *N* = 763 (ASD = 360; NT = 403) FCP: *N* = 828 (NT M = 357; NT F = 471)
Strategy 2 *Adjusting analytical pipelines*	*Z*-maps obtained after aligning processing with samples similar to strategy 1	Differences in MRI analytical methods (i.e., preprocessing and group model)	ABIDE I: *N* = 759 (ASD = 356; NT = 403) FCP: *N* = 824 (NT M = 356; NT F = 468)
Strategy 3 *Adjusting age*	*Z*-maps obtained in age-matched ABIDE I and FCP subsamples	Differences in sample age and MRI analytical methods	ABIDE I: *N* = 199 (ASD = 93; NT = 106) FCP: *N* = 439 (NT M = 183; NT F = 256)
Strategy 4 *Independent NT sample*	*Z*-maps of ABIDE I subsample as strategy 3 and an independent sex difference sample from Brain Genomics Superstruct Project (GSP)	Differences in sample age and MRI analytical methods	ABIDE I: *N* = 199 (ASD = 93; NT = 106) GSP: *N* = 742 (NT M = 320; NT F = 422)

*ABIDE* Autism Brain Imaging Data Exchange, *FCP* 1000 Functional Connectome Project, *GSP* Brain Genomics Superstruct Project, *ASD* autism spectrum disorder, *NT* neurotypicals, *M* males, *F* females

### Conjunction analyses

For all analyses across primary as well as validation and replication analytical strategies, we applied a voxel-level thresholding consistent with prior work [3, 33, 34]. *Z*-maps were voxel-level-thresholded across 500 successive thresholds ranging from *p* < 0.05 to *p* < 0.0001 decrementing by 0.0001. This multi-threshold approach illustrates whether findings are stable and consistent across thresholds. No cluster-level thresholds were applied, as different statistical comparisons in independent cohorts (ABIDE I and FCP) can result in different spatial extent thresholds [3, 33, 34]. Spatial overlap was obtained by conjunction analyses of each voxel-level thresholded *Z*-maps with logical “AND” masking [35]. The extent of overlap for each conjunction contrast was quantified as the average of the proportion of the total number of suprathreshold voxels for each map (Additional file 2: Supplementary methods) [3, 33, 34].

We then verified that spatial overlaps were not random by contrasting them against a null distribution of random overlaps derived from Monte Carlo simulations with 5000 iterations, for all successive 500 voxel-level thresholds (*p* = 0.05 to *p* = 0.0001) [3, 34, 36]. For each iteration, two whole gray matter maps were created, with the same voxel resolutions as the observed maps filled with values randomly sampled from a Gaussian distribution. Their spatial smoothness was then adjusted based on the average smoothness across the two observed maps. This estimation was done using the AFNI command *3dFWHMx*, and resulting values were averaged across ABIDE I and FCP, and across ABIDE I and GSP. Simulated maps were also thresholded along 500 voxel-level thresholds as the observed maps, and their overlap percentage was calculated. For each R-fMRI metric, further inferences were based on the spatial overlaps consistently above the 99.5th percentile of the null distribution for at least 70% of the 500 tested thresholds (i.e., at least 350 out of 500 instances). All computations were run with MATLAB version 2013a (The MathWorks Inc., Natick, MA, USA).

### Spatial overlap scenarios

**Fig. 1:**
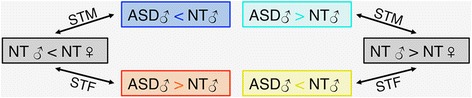
Spatial overlap scenarios. Depending on the combination of statistical contrasts being overlapped, four different scenarios can emerge. Decreases or increases of R-fMRI metrics in males with autism spectrum disorder (ASD) relative to neurotypical males (NT M) lead to different scenarios depending on whether they overlap with increases or decreases in NT M relative to NT females (F) (gray boxes). Following the predictions of the Extreme Male Brain (EMB) theory and Gender Incoherence (GI) theory, two scenarios fit the EMB model predicting a shift-towards-maleness (STM) in males with ASD (see blue and turquois boxes) and two scenarios fit the GI model predicting a shift-towards-femaleness (STF) in males with ASD (see orange and yellow boxes)

Across all analytical strategies, spatial overlaps between ASD-related atypicality *Z*-maps and typical sex difference *Z*-maps may underlie distinct scenarios depending on the directionality of the contrasts utilized—i.e., ASD♂ > NT♂ and ASD♂ < NT♂ and NT♂ > NT♀ and NT♂ < NT♀. As illustrated in Fig. 1, four scenarios could emerge; two met the EMB predictions and two met the GI predictions, as detailed below.

1. Shift-towards-maleness (STM) ASD-related increases (*EMB 1*; ASD♂ > NT♂ and NT♂ > NT♀): Males with ASD have higher values on examined R-fMRI metrics (i.e., higher iFC in ReHo, PCC-iFC, DC, and VMHC and higher regional amplitude in fALFF) than typical males, and typical males have higher values than typical females;
2. Shift-towards-maleness (STM) ASD-related decreases (*EMB 2*; ASD♂ < NT♂ and NT♂ < NT♀): Males with ASD have lower values on examined R-fMRI metrics than typical males, and typical males have lower values than typical females;
3. Shift-towards-femaleness (STF) ASD-related increases (*GI 1*; ASD♂ > NT♂ and NT♂ < NT♀): Males with ASD have higher values on examined R-fMRI metrics than typical males, and typical males have lower values than typical females;
4. Shift-towards-femaleness (STF) ASD-related decreases (*GI 2*; ASD♂ < NT♂ and NT♂ > NT♀): Males with ASD have lower values on examined R-fMRI metrics than typical males, and typical males have higher values than typical females.

### Primary analysis (strategy 1): originally published *Z*-maps

Statistical *Z*-maps were obtained from previously published analyses in Di Martino et al. [1] for the ASD-related difference male sample (ABIDE I) and Yan et al. [19] for the typical sex difference sample (FCP). The ABIDE I study included 763 males (360 ASD♂ and 403 NT♂; aged 6–58 years). The typical sex difference study (FCP), which included 828 NT selected from the 1000 Functional Connectome Project, comprised of 357 NT♂ and 471 NT♀ between 8 and 78 years of age (Table 2). Details of data acquisition can be found on the ABIDE I and FCP websites, http://fcon_1000.projects.nitrc.org/indi/abide and http://fcon_1000.projects.nitrc.org/fcpClassic/FcpTable.html, respectively, and a summary of the analyses and results can be found in Additional files 2 and 3: Supplementary methods and results.

**Table 2:**
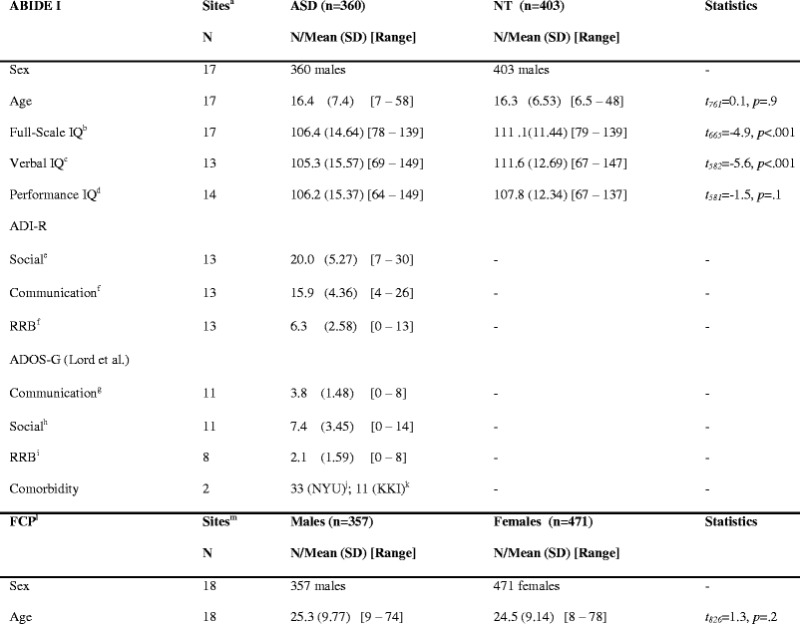
Characterization of the original ABIDE I and FCP samples

^a^ABIDE I data collections (sites): KKI, Leuven 1, Leuven 2, MaxMun, NYU, OHSU, OLIN, PITT, SDSU, Stanford, Trinity, UCLA 1, UCLA 2, UM 1, UM 2, USM, and Yale

^b^FIQ was available for 354 individuals with ASD and 401 neurotypical (NT) controls

^c^VIQ was available for 302 individuals with ASD and 324 NT

^d^PIQ was available for 304 individuals with ASD and 337 NT

^e^ADI-R Social scale score was available for 245 individuals with ASD

^f^ADI-R Communication and ADI-R restricted repetitve bevhavior scale scores were available for 246 individuals with ASD

^g^ADOS-G Communication scores were available for 238 individuals with ASD

^h^ADOS-G Social scores were available for 261 individuals with ASD

^i^ADOS-G RRB scores were available for 202 individuals with ASD

^j^Attention deficit hyperactivity disorder (ADHD; *N* = 10), oppositional defiant disorder (ODD; *N* = 1), mood disorder (*N* = 10), anxiety disorders (*N* = 11)

^k^ADHD (*N* = 6), ODD (*N* = 2), mood disorder (*N* = 1), anxiety disorders (*N* = 2)

^l^There was no information available on IQ in the FCP sample

^m^FCP data collections: Baltimore, Bangor, Beijing, Berlin, Cambridge, Cleveland, ICBM, Leiden 1, Leiden 2, Leipzig, Munchen, Newark, New York 1, New York 2, Orangeburg, Oulu, Queensland, and Saint Louis

### Validation analysis (strategy 2): adjusting for differences in analytical pipelines

To account for possible confounds in the primary analyses (strategy 1) due to partially different preprocessing pipelines and statistical group models between ABIDE I and FCP studies, both original datasets were reanalyzed using version 0.3.9.1 of the Configurable Pipelines for the Analysis of Connectomes (C-PAC) [37]. Briefly, nuisance regression included white matter and cerebrospinal fluid signals, 24 motion parameters based on Friston 24-Parameter Model [38], and linear and quadratic trends. All derivatives were smoothed by a 6-mm FWHM Gaussian kernel. Statistical *Z*-maps were generated within study-specific functional volume masks including voxels (in MNI space) present across all subjects in a given study sample (ABIDE I and FCP). Group-level analyses were conducted for each sample separately using a general linear model including diagnosis (ABIDE I) or sex (FCP) as the regressor of interest and age, site, mean framewise displacement [39], and individual subject means of each R-fMRI derivative as the nuisance covariates (Additional file 2: Supplementary methods). We then used the resulting *Z*-maps for voxel-level conjunction analyses as detailed above.

### Validation analysis (strategy 3): age-matched samples

To account for differences in age distribution between the ABIDE I and FCP samples, we applied the same analytical pipeline used for strategy 2 on subsamples matched for age (range 17.5–37 years). The age-matched ABIDE I sample comprised 199 individuals (93 ASD♂; 106 NT♂), whereas the age-matched FCP sample included 439 neurotypical individuals (183 NT♂; 256 NT♀) (Additional file 4: Figure S2 and Additional file 5: Table S1). Group analyses were conducted as described in strategy 2. We then used the resulting *Z*-maps for voxel-level conjunction analyses as detailed above.

### Replication analysis (strategy 4): replication with an independent typical sex differences sample

Data were obtained from the GSP [20], matched for age based on the same criteria of strategy 3, resulting in a sample of 742 individuals comprising 320 NT♂ and 422 NT♀ ranging between 18 and 35 years of age (Additional file 5: Table S1). Analyses were conducted as in strategy 2. We then used the resulting *Z*-maps for voxel-level conjunction analyses as detailed above.

## Results

### Overview

**Fig. 2:**
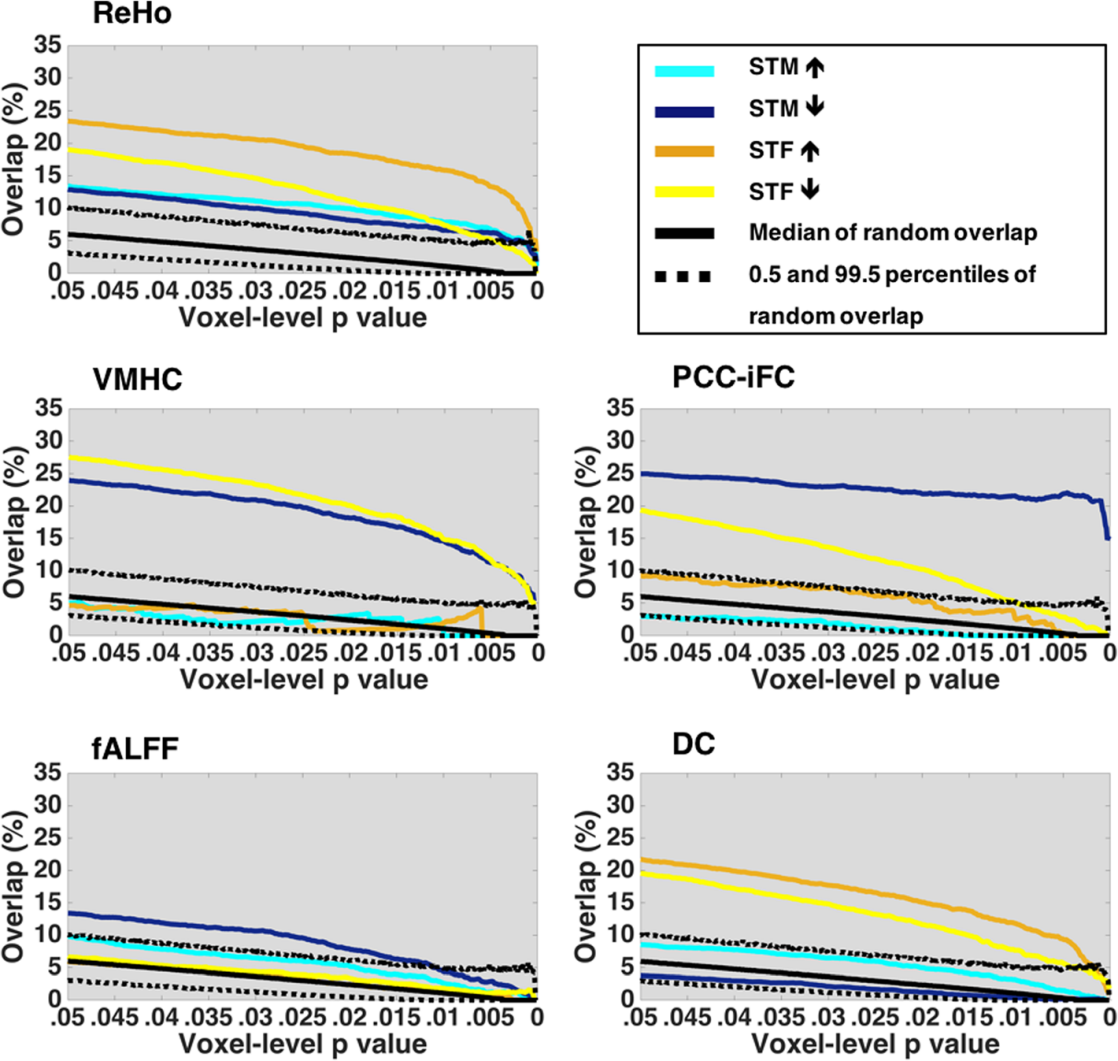
Conjunction analyses. Plots (one per each resting-state fMRI metric [R-fMRI]) show the spatial overlap percentages across 500 successive statistical voxel-level thresholds for the four possible overlap scenarios (turquoise: shift-towards-maleness (STM) ASD-related increases (EMB 1; as indicated by the upward arrow) = ASD♂ > NT♂ and NT♂ > NT♀; blue: STM ASD-related decreases (EMB 2; as indicated by the downward arrow) = ASD♂ < NT♂ and NT♂ < NT♀; orange: shift-towards-femaleness (STF) ASD-related increases (GI 1; as indicated by the upward arrow) = ASD♂ > NT♂ and NT♂ < NT♀; yellow: STF ASD-related decreases (GI 2; as indicated by the downward arrow) = ASD♂ < NT♂ and NT♂ > NT♀). The black solid line represents the median of the null distribution of the random spatial overlap generated by 5000 Monte Carlo simulations for each threshold. The dotted lines mark the 0.5th and 99.5th percentiles of the null distribution of random spatial overlap. Only spatial overlaps consistently above the 99.5th percentile of the null distribution for at least 70% of the 500 tested thresholds were utilized for subsequent results’ characterization. R-fMRI abbreviations: *DC* degree centrality, *fALFF* fractional amplitude of low frequency fluctuations, *ReHo* regional homogeneity, *VMHC* voxel-mirrored homotopic connectivity, *PCC-iFC* posterior cingulate cortex intrinsic functional connectivity

**Fig. 3:**
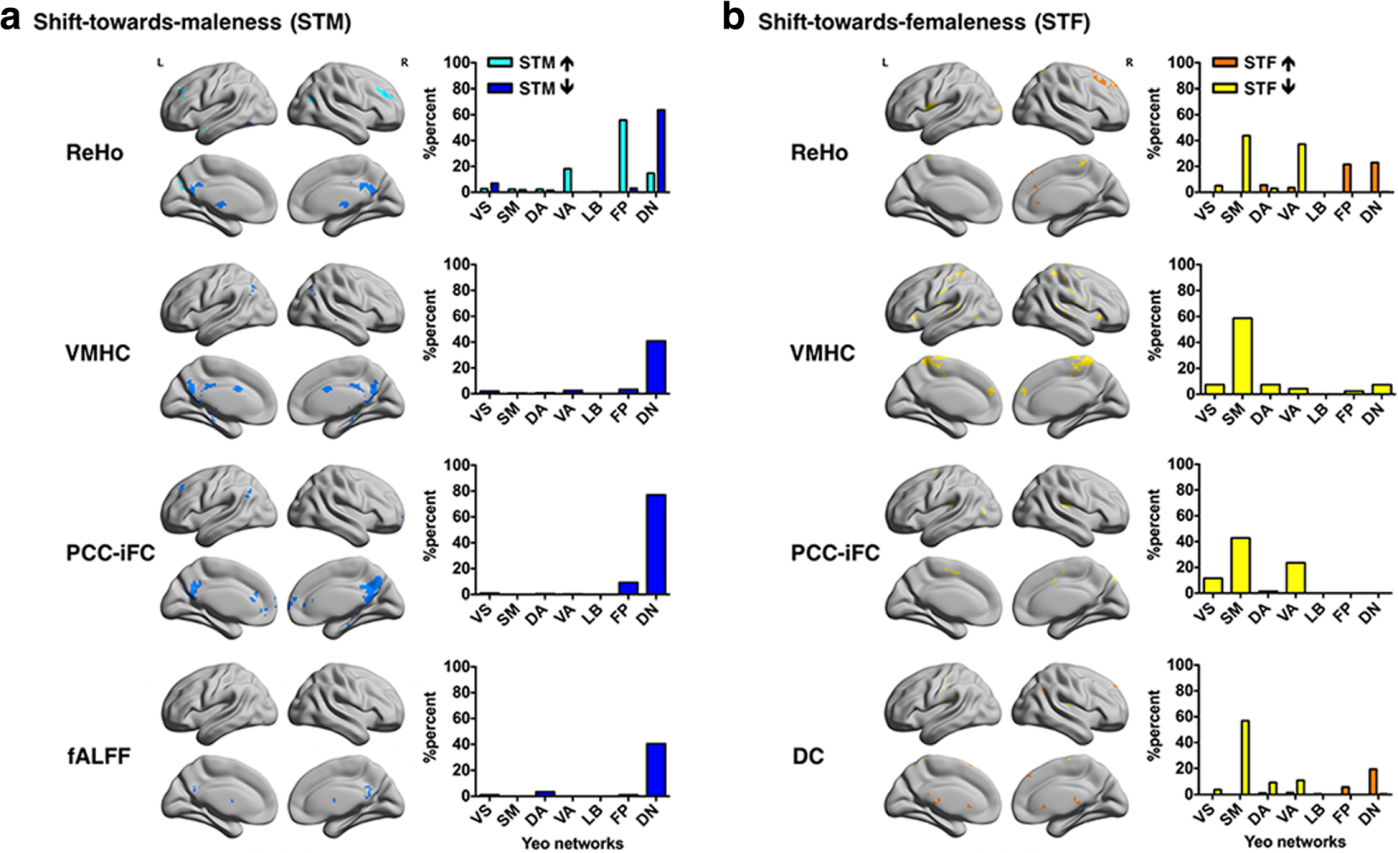
Overlaps consistent with a shift-towards-maleness and a shift-towards-femaleness. For each panel, **a** and **b**, the left columns illustrate on inflated surface maps (BrainNet Viewer; https://www.nitrc.org/projects/bnv) the regions of significant spatial overlap based on the conjunction of statistical *Z*-maps resulting from the ABIDE I and FCP studies (voxel-level thresholded at *Z* ≥ 2.58) for each resting-state fMRI (R-fMRI) metric. The histograms in the right column of each panel describe the percentage of voxels within the above clusters included in the seven functional cortical networks described by Yeo et al. [40]. R-fMRI abbreviations: *DC* degree centrality, *fALFF* fractional amplitude of low frequency fluctuations, *ReHo* regional homogeneity, *VMHC* voxel-mirrored homotopic connectivity, *PCC-iFC* posterior cingulate cortex intrinsic functional connectivity. Seven functional Yeo networks: *VS* visual network, *SM* somatomotor network, *DA* dorsal attention network, *VA* ventral attention network, *LB* limbic network, *FP* fronto-parietal network, *DN* default network. **a** Overlaps consistent with the Extreme Male Brain (EMB) theory: Hyper-connectivity consistent with a shift-towards-maleness (STM) was mainly present in the FP network for ReHo, whereas an STM hypo-connectivity for ReHo, VMHC, and PCC-iFC and decreased fALFF were mainly centered around the DN. Color codes: turquoise = STM ASD-related increases (EMB 1); blue = STM ASD-related decreases (EMB 2). **b** Overlaps consistent with the Gender Incoherence (GI) theory: Hyper-connectivity consistent with a shift-towards-femaleness (STF) was mainly in DN for DC and ReHo, whereas an STF hypo-connectivity across DC, ReHo, VMHC, and PCC-iFC was mainly centered around the SM network. Color codes: orange = STF ASD-related increases (GI 1); yellow = STF ASD-related decreases (GI 2)

Conjunction analyses revealed overlaps between intrinsic brain properties characterizing typical sex differences and those characterizing ASD vs. NT differences in males (Fig. 2). Examining overlaps along successive voxel-level thresholds showed that they were significant above the 99.5th percentile of the null distribution across all R-fMRI measures. Depending on the R-fMRI metric, on average, 13 to 31% of those voxels characterized by ASD-related differences overlapped with those characterized by typical sex differences (inter-quartile range across all 500 voxel-level thresholds = 25–35% for ReHo; 21–31% for PCC-iFC; 17–30% for DC; 13–21% for VMHC; 8–16% for fALFF). As detailed below, based on the directionality of the *Z*-map contrasts involved in these non-random overlaps, findings were consistent with GI and EMB in *distinct* functional regions. To functionally characterize these spatial overlaps, we mapped them into the seven functional cortical networks as described by Yeo et al. [40] (Additional file 2: Supplementary methods). A double dissociation emerged. As illustrated in the maps thresholded at *Z* ≥ 2.58 in Fig. 3, regions matching EMB predictions mainly involved DN and related circuits involving higher-order socio-emotional and cognitive control processes. In contrast, regions matching GI predictions mainly centered around somatomotor (SM) circuits. This overall pattern of results was robust to permutation testing used as an alternative approach to Monte Carlo simulations (for generating the null distribution of random overlaps), as reported in supplementary post hoc analyses (Additional file 6: Table S2).

### Shift-towards-maleness (EMB predictions)

A pattern of overlap consistent with a shift-towards-maleness (STM) characterized all R-fMRI metrics except DC (Fig. 2). Further, while only ASD-related increases in ReHo (EMB 1) significantly overlapped with typical sex differences, ASD-related decreases (EMB 2) did so for a wider range of R-fMRI metrics including ReHo, VMHC, PCC-iFC, and fALFF. These ASD-related decreases predominantly encompassed the DN—particularly the precuneus and PCC. The ASD-related hyper-connectivity in local iFC (i.e., ReHo) encompassed the fronto-parietal (FP) network and, to a lesser extent, the ventral attention (VA) network and DN including the frontal pole, middle frontal gyrus, and lateral occipital cortex (Fig. 3a and Additional file 7: Table S3).

### Shift-towards-femaleness (GI predictions)

A pattern of overlap consistent with a shift-towards-femaleness (STF) characterized all R-fMRI metrics, except fALFF. Significant overlaps with typical sex differences involved ASD-related increases (GI 1) in ReHo and DC and decreases (GI 2) in ReHo, DC, VMHC, and PCC-iFC (Fig. 2). ASD-related R-fMRI decreases predominantly encompassed the SM network and to a lesser degree the ventral, visual, and dorsal attention networks, mainly centering around the postcentral gyrus, central and parietal operculum, and Heschl gyrus. ASD-related hyper-connectivity encompassed the DN and, to a lesser extent, the FP network, across DC and ReHo (Fig. 3b and Additional file 7: Table S3).

### Cognitive ontology

**Fig. 4:**
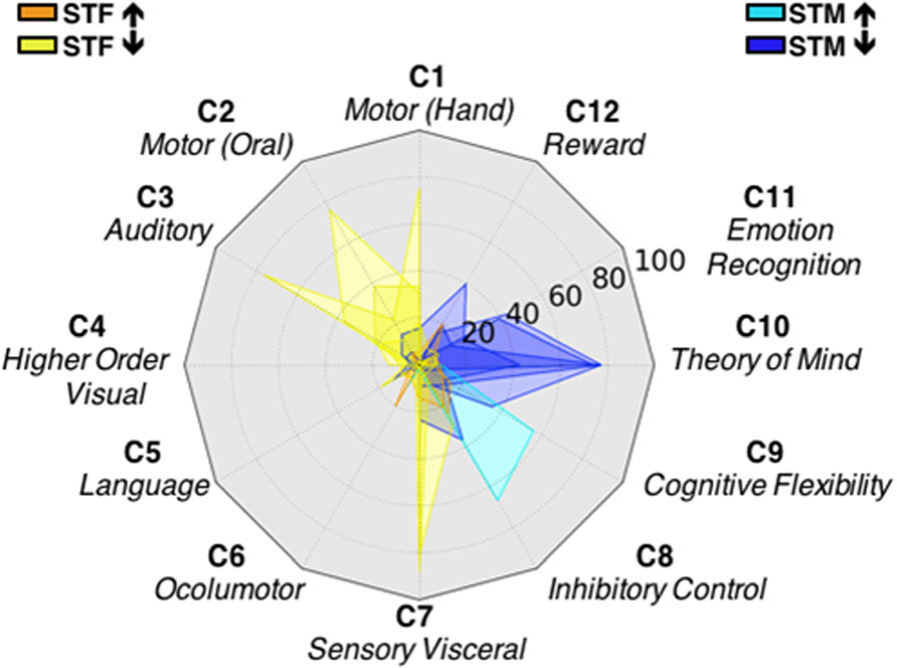
Cognitive ontology maps. The polar plot shows the percentage (0–100%) of overlap between the significant conjunctions of statistical *Z*-maps (voxel-level thresholded at *Z* ≥ 2.58) and the 12 Yeo cognitive ontology probability maps [41] (probability thresholded at *p* = 1e−5) for cognitive components C1–C12. We labeled each component based on the top five tasks reported to be most likely recruited by a given component. Results are summarized according to their consistency with the Extreme Male Brain (EMB) or Gender Incoherence (GI) models, regardless of the resting-state fMRI metric. Color codes: turquoise = shift-towards-maleness (STM) ASD-related increases (EMB 1); blue = STM ASD-related decreases (EMB 2); orange = shift-towards-femaleness (STF) ASD-related increases (GI 1); yellow = STF ASD-related decreases (GI 2)

To explore the cognitive domains implicated in the above regions, we quantified the percentage of their overlap with the 12 cognitive ontology maps by Yeo et al. [41] thresholded at *p* = 1e−5. We labeled each component based on the top five tasks it recruits. Consistent with the above functional network spatial distribution of our findings, a double dissociation was evident. In males with ASD, findings consistent with a shift-towards maleness involved higher-order social and cognitive processes, while those consistent with a shift-towards-femaleness mostly involved lower-order sensory and motor processes (Fig. 4 and Additional file 8: Table S4).

### Validation and replication analyses

Results were highly similar across strategies, albeit greatest similarity was observed when analytical pipelines and age were aligned, i.e., strategies 3 and 4 (Additional files 9, 10, 11, and 12: Figures S3–S6 and Additional file 13: Table S5). A shift-towards-maleness in ASD-related decreases in PCC-iFC and VMHC was the most consistent findings across strategies, followed by those observed for ASD-related decreases in ReHo and, to a lesser extent, fALFF in DN (EMB 2) (Additional file 14: Table S6). For further details, see Additional file 3: Supplementary results.

## Discussion

To characterize the neurophenotypic convergence of ASD-related intrinsic brain characteristics and typical sex differences, we examined the spatial overlap between neurotypical sex-related and ASD-related intrinsic brain properties in large independent samples [1, 19]. Results provided insights into two competing models of such convergence predicting either a shift-towards-maleness (EMB) [16] or a shift-towards-femaleness (GI) [18] in males with ASD. Across R-fMRI metrics, analyses revealed evidence consistent with both models, yet involving distinct functional neural networks. A shift-towards-maleness in males with ASD mostly involved DN and FP networks serving higher-order socio-emotional and cognitive control processes. In contrast, a shift-towards-femaleness in males with ASD predominantly centered around the SM network. These patterns remained stable across analytical strategies adjusting for differences in preprocessing pipelines and samples. The results suggest that previously reported R-fMRI abnormalities in males with ASD may partly result from atypical sexual differentiation in the brain, and these mechanisms act in a network-specific manner.

In typical individuals, a mosaic of brain region-specific masculinization and feminization exists across the sexes [42–45]. Our findings of coexisting shift-towards-maleness and shift-toward-femaleness of intrinsic brain properties in males with ASD suggest that biological mechanisms involved in sex mosaicism partly contribute to the neural characteristics of ASD. These may involve hormonal and non-hormonal factors [44, 46]. For example, estradiol can induce opposite effects in distinct brain regions by either initiating or preventing cell death and synaptogenesis, as well as by enhancing or dampening excitation [47]. Beyond hormonal factors, brain regional sexual differentiation is also driven by sex differential gene expression [46, 48]. Recent studies have shown that male differential expression of astrocyte and microglial genes are upregulated in ASD [13]. How these molecular phenomena affect the macro-scale intrinsic functional brain organization should be the focus of multilevel approaches that generate a unifying model of the relation between typical sex differentiation and ASD.

In regard to the macro-scale networks and functional processes involved, our most consistent finding was the shift-towards-maleness of DN, a network widely implicated in ASD [11, 23, 49–51]. In line with prior work [10, 11], we found that significant overlaps with typical sex differences encompassed PCC-iFC decreases along the DN midline. By extending our exploration to the whole brain, analyses revealed similar shift-towards-maleness exist for a range of intrinsic properties in the PCC. These included ASD-related decreases in local connectivity (ReHo) [52], homotopic inter-hemispheric interactions (VMHC) [30], and fractional amplitude of low frequency fluctuations (fALFF) [32]. These findings suggest that examinations of DN in ASD should consider sex-dependent biological factors.

Beyond DN ASD-related R-fMRI decreases, a shift-towards-maleness also involved other processes, especially ASD-related increases in FP local connectivity (ReHo). Notably, DN and FP networks subserve higher-order processes that are core to EMB theory’s postulation that individuals with ASD are weaker “mentalizers” and stronger “systemizers” [16, 17, 53]. Consistent with the DN’s role in social cognition and mentalizing, the shift-towards-maleness of atypical intrinsic properties in ASD mapped onto cognitive components associated with theory of mind and emotion recognition processes. Impairments in these domains are referred to as atypical mentalizing and characterize individuals with ASD [53]. On the other end, the FP network mapped onto inhibitory control and cognitive flexibility. Impairments in these domains have been attributed to weak central coherence, perseveration, and hyper-systemizing in ASD [54].

Our systematic examination of the intrinsic functional brain also revealed evidence of a shift-towards-femaleness in the male ASD brain in the SM network comprising motor and auditory cortices. Altered sensory-motor processing has often been observed in ASD [55, 56], and associated atypical intrinsic brain properties are emerging [57–61]. Our findings suggest that a biological shift-towards-femaleness in the SM network may underlie these atypicalities [62, 63]. Alternatively, a SM shift-towards-femaleness might result from experience (e.g., being less engaged in motor activities). Longitudinal studies are required to clarify the impact of experiential factors [64], sex-specific biological factors, and their interactions. Finally, although in NT individuals motor and language processes are hemispherically specialized, NT females have greater bi-hemispheric integration compared to NT males [65]. Given prior reports of a reduction of typical asymmetries in males with ASD [58, 66, 67], our findings of a shift-towards-femaleness involving motor and auditory domains in males with ASD suggest that biological sex-related factors are likely involved in atypical inter-hemispheric interactions in this population.

With respect to specific R-fMRI features and atypical ASD-related differences, we note that shift-towards-femaleness or shift-towards-maleness in ASD did not affect any of the metrics differentially. Instead, echoing recent large-scale studies reconciling prior mixed findings of hypo- and hyper-connectivity in ASD [1, 24], results varied by the functional network involved. Our findings further suggest that sex-related biological factors contribute to the complex presentation of atypical iFC in ASD.

Results should be interpreted considering several limitations. First, a sufficiently large dataset was only available for males with ASD. While focusing on a large ASD male sample allowed us to address prior inconsistencies about a shift-towards-femaleness in males with ASD [10, 11], future large-scale characterizations of both females and males will provide insights into the role of typical sexual differentiation in ASD for both sexes [14, 68]. Second, while patterns of shift-towards-maleness and shift-towards-femaleness were similar across analytical strategies, aspects of the shift-towards-femaleness were more variable between the primary strategy and those adjusting for differences in age range. Speculatively, the shift-towards-maleness in ASD may therefore be related to *organizational* effects on neurodevelopment, as predicted by the EMB model that posits elevated prenatal steroidogenic processes [16, 69, 70]. An ASD-related shift-towards-femaleness in males could reflect later events even during and beyond puberty. This would be in line with the GI model which was mostly conceptualized based on postpubertal physiological measures [18]. As such, a shift-towards-femaleness may be more variable depending on the sample age. A cross-sectional examination of age effects would require a larger and more homogenous age distribution across data acquisition sites than the present one. Nevertheless, as we included age as a nuisance covariate in the statistical models used to generate the *Z*-maps overlapped, potential confounds on the present results are limited. Third, it was not possible to address the role of comorbid psychiatric conditions in our findings due to limited availability of this information across the ABIDE I datasets. Many comorbid conditions within ASD, such as attention deficit hyperactivity disorder, show a sex-biased prevalence ratio themselves [71, 72]. Further, recent studies considering comorbidities in individuals with ASD show brain connectivity patterns that are specific to ASD comorbidities [27]. This calls for characterization of both ASD core and comorbid symptoms in neuroimaging studies of ASD. Finally, given that FCP and ABIDE I aggregate data retrospectively across multiple sites, unknown confounds due to site differences may exist. We limited this concern by including sites as covariates at the group-level comparisons generating *Z*-maps used to assess spatial overlaps.

## Conclusion

In conclusion, biological factors involved in typical sex differentiation are likely to affect the intrinsic functional properties of the male ASD connectome and manifest in both shift-towards-maleness and shift-towards-femaleness in different neural networks. The present findings suggest that a model based on network-dependent atypical sex mosaicism can synthesize seemingly competing EMB and GI theories. Given the heterogeneity of ASD, future studies combining multidimensional indices of shift-towards-maleness and shift-towards-femaleness and data-driven clustering methods can assess the extent to which sex mosaicism varies across individuals and may identify subgroups of ASD with different biological underpinnings.

## Additional files

Additional file 1: ABIDE and FCP results. (TIFF 3571 kb)

Additional file 2: Supplementary methods. (DOCX 149 kb)

Additional file 3: Supplementary results. (DOCX 120 kb)

Additional file 4: Age Matching. (TIFF 3751 kb)

Additional file 5: Characterization of age-matched ABIDE I, FCP and GSP samples. (DOCX 27 kb)

Additional file 6: Comparison of percentage of overlaps at Z ≥ 2.58 for the real overlap, random overlap generated by 5000 Monte Carlo simulation, and random overlap generated by 1000 permutations, across R-fMRI metrics by model. (DOCX 147 kb)

Additional file 7: Percentages of voxels within the conjunction maps (thresholded at Z ≥ 2.58) overlapping with the seven functional cortical networks per Yeo et al. [40]. (DOCX 139 kb)

Additional file 8: Percentage of voxels within the conjunction maps (thresholded at Z ≥ 2.58) and the 12 cognitive ontology maps defined Yeo et al. [41] (probability thresholded at p = 1e-5). (DOCX 178 kb)

Additional file 9: Conjunction Analyses Across Strategies 2-4. (TIFF 3298 kb)

Additional file 10: Overlaps Consistent with a Shift-Towards-Maleness (EMB) across Strategies 2–4. (TIFF 2666 kb)

Additional file 11: Overlaps Consistent with a Shift-Towards-Femaleness (GI) across Strategies 2–4. (TIFF 2628 kb)

Additional file 12: Cognitive Ontology Maps for Strategies 2-4. (TIFF 1438 kb)

Additional file 13: Similarity across analytical strategies. (DOCX 61 kb)

Additional file 14: Similarity across R-fMRI metrics by model across all analytical strategies. (DOCX 80 kb)

## Abbreviations

ABIDE: Autism Brain Imaging Data Exchange
ASD: Autism spectrum disorder
DC: Degree centrality
DN: Default network
EMB: Extreme Male Brain theory
fALFF: Fractional Amplitude of Low Frequency Fluctuations
FCP: 1000 Functional Connectome Project
FP: Fronto-parietal
GI: Gender Incoherence theory
GSP: Brain Genomics Superstruct Project
NT F: Neurotypical females
NT M: Neurotypical males
PCC-iFC: Posterior cingulate cortex intrinsic functional connectivity
ReHo: Regional homogeneity
SM: Somatomotor
STF: Shift-towards-femaleness
STM: Shift-towards-maleness
VMHC: Voxel-mirrored homotopic connectivity
