# Supplementary figures and images for: Network-specific sex differentiation of intrinsic brain function in males with autism
Source: Mol Autism. 2018 Mar 6;9:17. doi: 10.1186/s13229-018-0192-x (PMC5840786; doi:10.1186/s13229-018-0192-x)

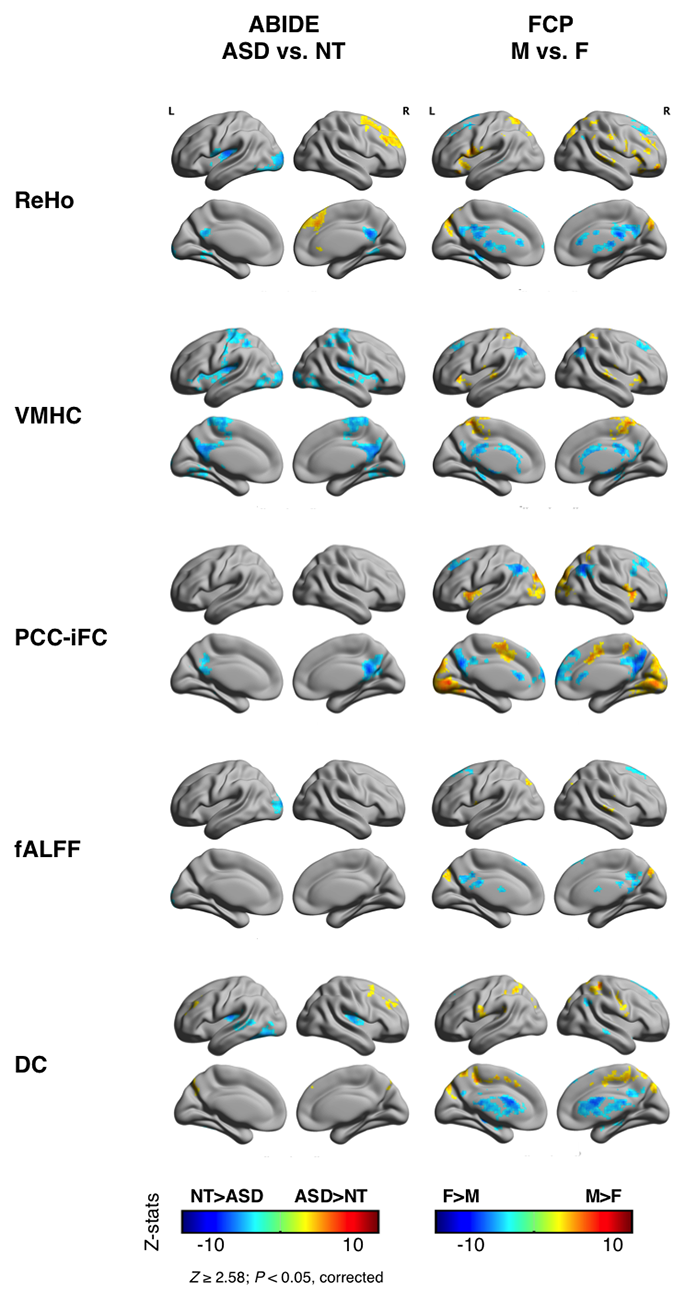

Supplement: Supplementary file 1 — ABIDE and FCP results. (TIFF 3571 kb) [file 13229_2018_192_MOESM1_ESM.tif]

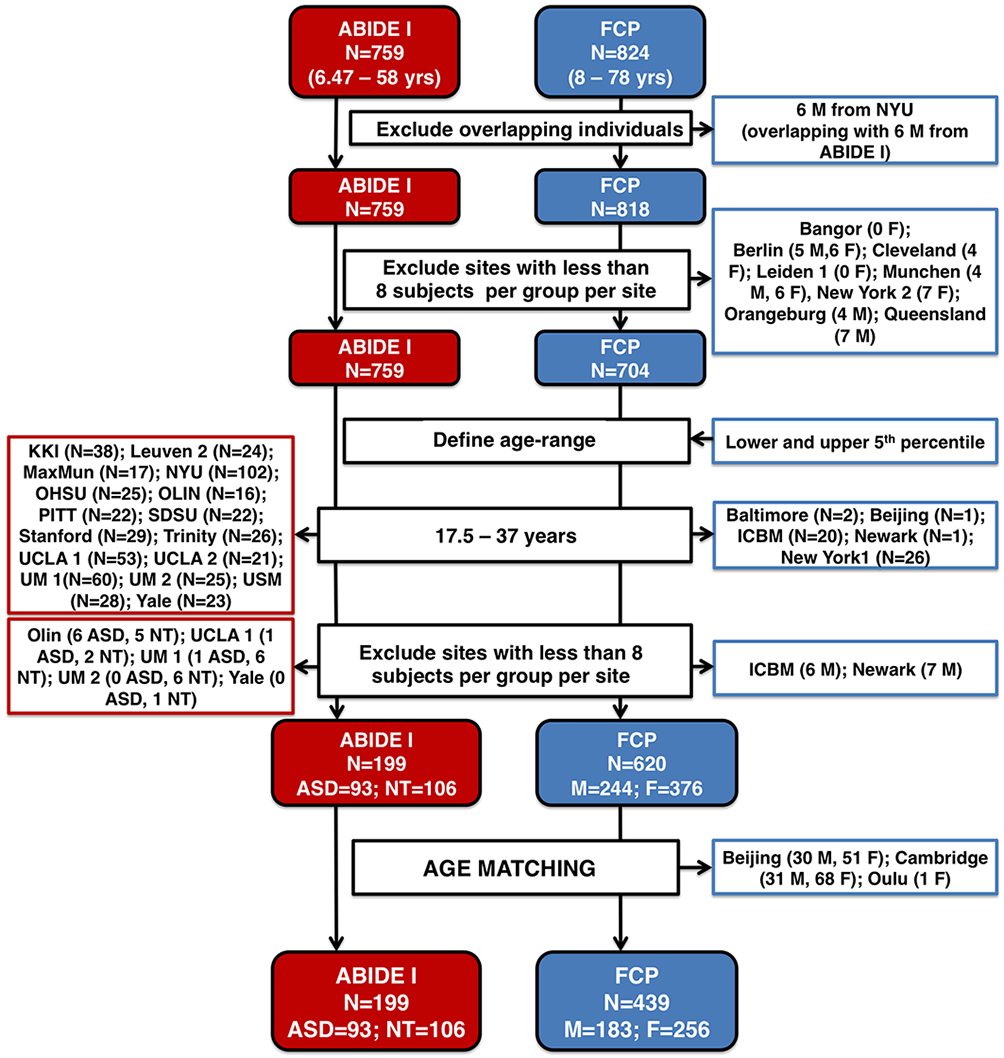

Supplement: Supplementary file 4 — Age Matching. (TIFF 3751 kb) [file 13229_2018_192_MOESM4_ESM.tif]

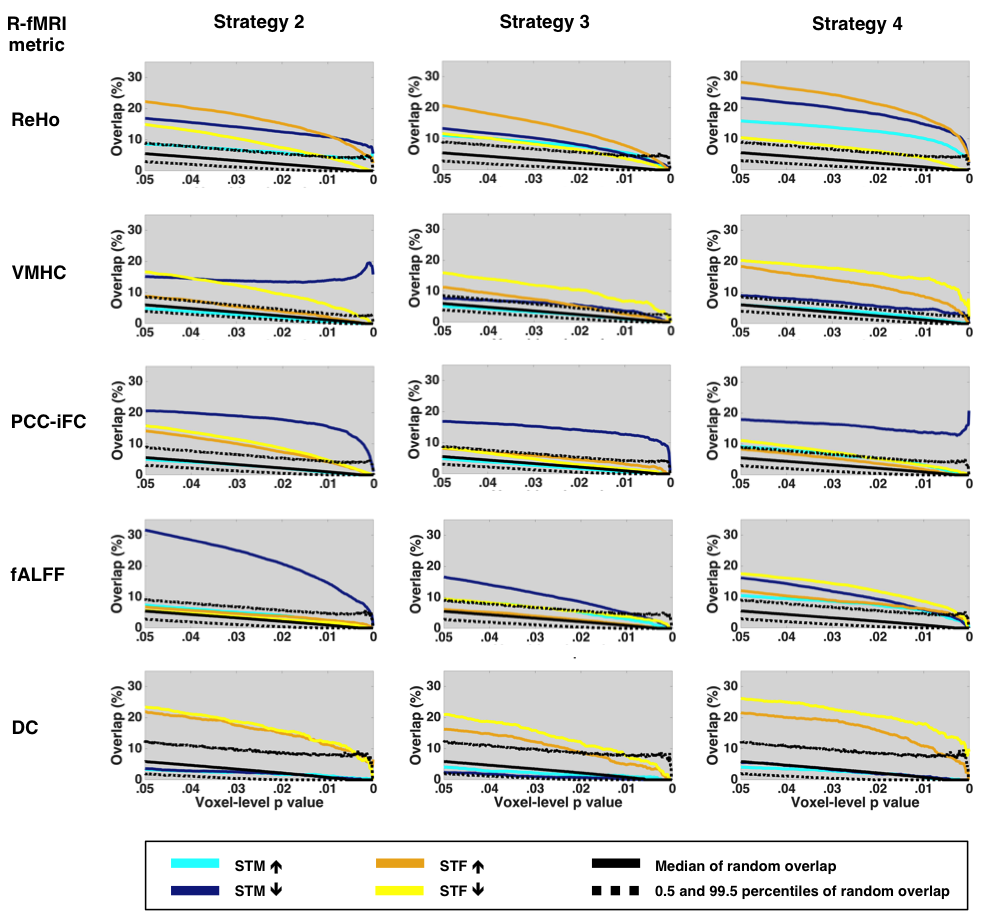

Supplement: Supplementary file 9 — Conjunction Analyses Across Strategies 2-4. (TIFF 3298 kb) [file 13229_2018_192_MOESM9_ESM.tif]

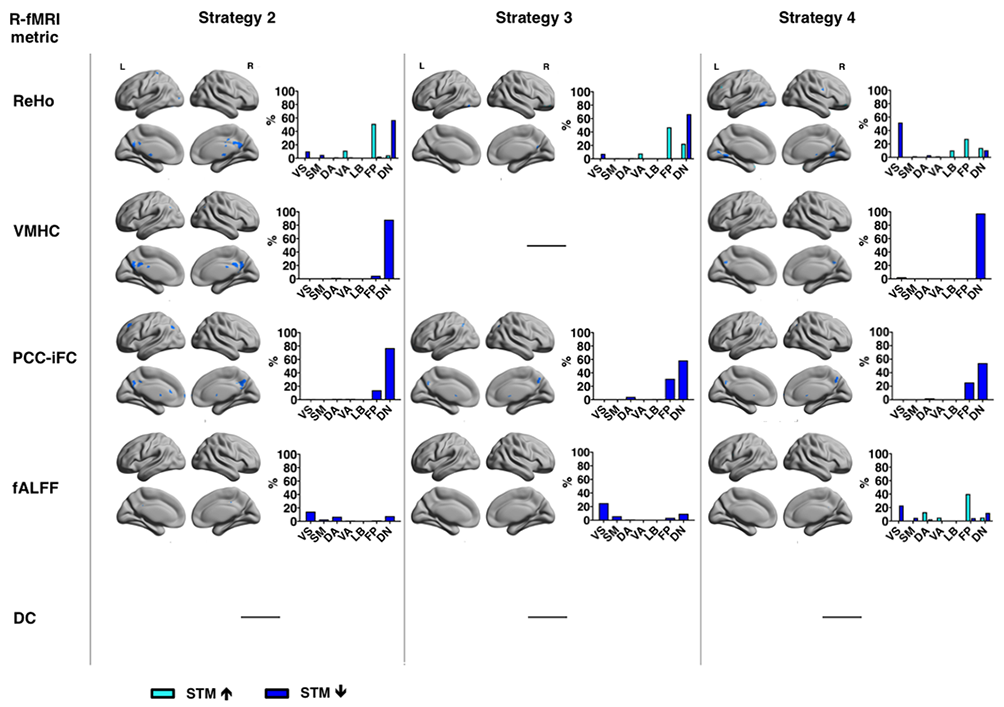

Supplement: Supplementary file 10 — Overlaps Consistent with a Shift-Towards-Maleness (EMB) across Strategies 2–4. (TIFF 2666 kb) [file 13229_2018_192_MOESM10_ESM.tif]

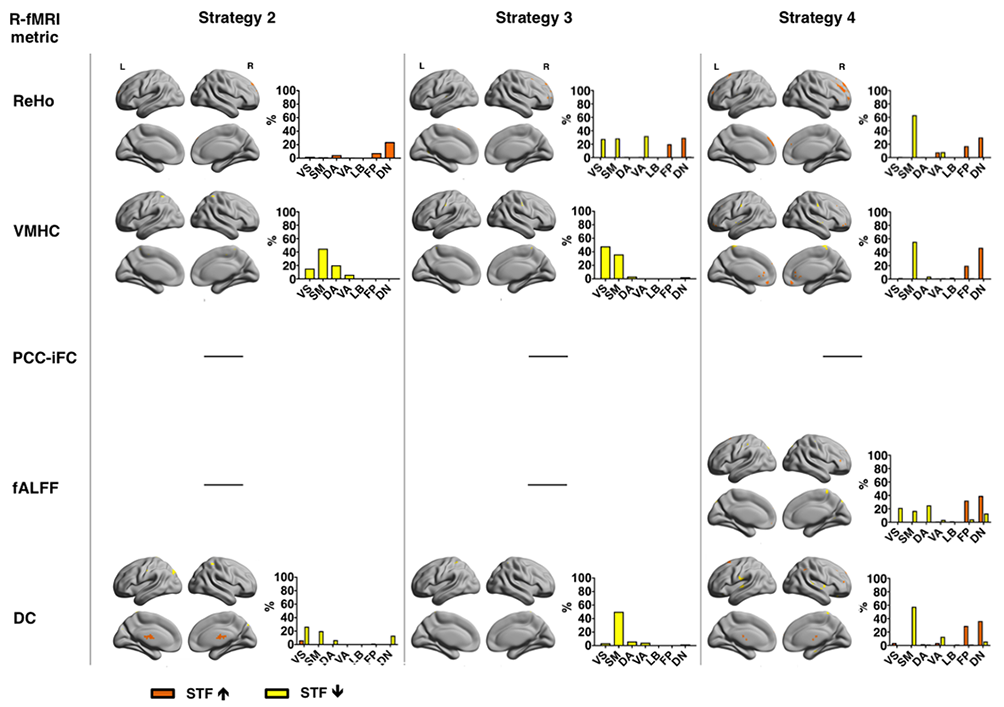

Supplement: Supplementary file 11 — Overlaps Consistent with a Shift-Towards-Femaleness (GI) across Strategies 2–4. (TIFF 2628 kb) [file 13229_2018_192_MOESM11_ESM.tif]

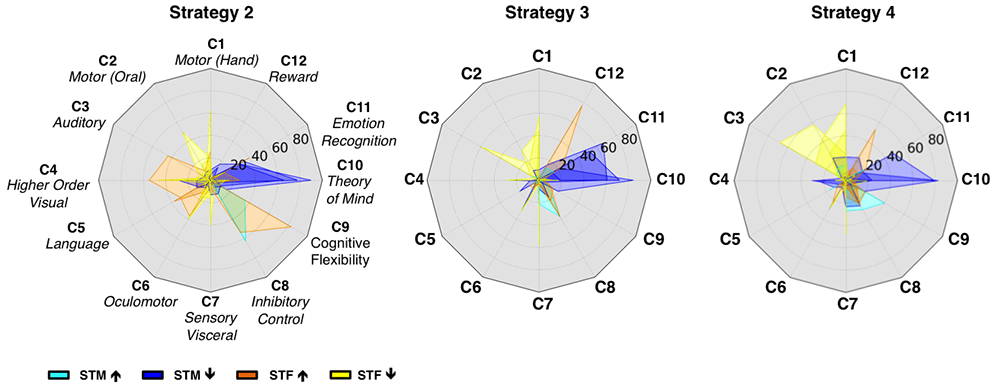

Supplement: Supplementary file 12 — Cognitive Ontology Maps for Strategies 2-4. (TIFF 1438 kb) [file 13229_2018_192_MOESM12_ESM.tif]
