# Supplementary material for: Network-specific sex differentiation of intrinsic brain function in males with autism
Source: Mol Autism. 2018 Mar 6;9:17. doi: 10.1186/s13229-018-0192-x (PMC5840786; doi:10.1186/s13229-018-0192-x)
Supplement: Supplementary file 2 — Supplementary methods. (DOCX 149 kb) [file 13229_2018_192_MOESM2_ESM.docx]

**Additional file 2: Supplementary methods**

**Overview**

Using conjunction analyses, we explored whether typical sex differences in intrinsic brain properties overlapped with those characterizing males with Autism Spectrum Disorder (ASD) vs. neurotypical (NT) males. We used four analytical strategies. In our primary analysis (**Strategy 1**) we overlapped the original statistical Z-maps resulting from two previously published resting-state functional magnetic resonance imaging (R-fMRI) studies [1, 2]. One map represented typical sex difference based on data from the Functional Connectome Project (FCP) repository [2], the other represented atypical intrinsic functional properties in males with ASD vs. NT using an Autism Brain Imaging Data Exchange (ABIDE I) repository [1]. See Additional file 1: Fig. S1 for an illustration of the group comparison *Z*-maps, here thresholded at voxel-level *Z* ≥ 2.58 and *p* < 0.05 at the cluster level.

In secondary strategies (**Strategies 2 to 4**), we repeated conjunction analyses after addressing possible confounds introduced by differing analytical methods between the two studies/samples. We did so with and without adjusting for differences in mean age between the two studies/samples (**Strategies 2 and 3**, respectively). Finally, we examined the consistency of findings by repeating conjunction analyses using an independent typical sex differences sample obtained from the Brain Genomics Superstruct Project (GSP) [3] using the same analytical pipeline and age-matched ASD subsamples as in Strategies 2 and 3 (**Strategy 4**). Five different R-fMRI metrics were investigated: 1) regional homogeneity (ReHo) [4], 2) voxel-mirrored homotopic connectivity (VMHC) [5], 3) network degree centrality (DC) [6], 4) posterior cingulate cortex-based intrinsic functional connectivity (PCC-iFC) and 5) fractional amplitude of low frequency fluctuations (fALFF) [7].

**R-fMRI measures**

*Regional Homogeneity (ReHo)* [4] is a measure of regional coherence between neighboring fMRI time series. It is based on the Kendall’s coefficient of concordance [8] between a voxel’s time series and its 26 adjacent neighbors. Subject-level maps were transformed into subject-level Z-score maps.

*Voxel-Mirrored Homotopic Connectivity (VMHC)* [5] is the Pearson’s correlation between each voxel and its geometrically corresponding symmetric counterpart in the opposite hemisphere. Spatial transformation parameters were based on a registration to a symmetric MNI template to increase spatial correspondence between homotopic voxels. Correlation coefficients were standardized by applying a Fisher’s *r*-to-*Z*-transformation.

*Network Degree Centrality (DC)* [6] is a measure of local network connectivity. To be consistent with the studies included in our primary analyses [1, 2], here, it is based on a given voxel’s sum of significant connections with corresponding *p* < 0.001. DC was calculated based on a study-specific functional volume mask based on voxels (in MNI space) present in at least 90% of subjects and further constrained by a 25% gray matter (GM) probability mask. Voxel-size was down-sampled to 4mm^3^ to reduce computational intensity. Voxel-based graphs were then generated by computing the Pearson’s correlation of each voxel’s extracted time series with every other voxel’s extracted time series within the study-specific mask. A significance threshold of *p* < 0.001 was applied resulting in a binary, undirected adjacency matrix. DC was then computed by counting the number of significant connections in the adjacency matrix. Subject-level DC-maps were standardized using *Z*-score transformations.

*Seed-based Correlation Analysis* (SCA) was carried out by extracting the mean time series from a spherical region-of-interest mask centered in PCC. Since the original FCP and ABIDE I studies slightly differed in the PCC seed location [FCP: x=0, y=-53, z=26, [9]; 10mm diameter sphere; ABIDE I: x=8, y=-56, z=26, 8mm diameter sphere, 10], all secondary analyses (Strategies 2-4) harmonized preprocessing using the FCP PCC coordinates for an 8mm diameter spherical mask. Pearson’s correlation coefficient was calculated between the PCC time series and each voxel in the brain before being Fisher’s *z*-transformed.

*Fractional Amplitude of Low Frequency Fluctuations (fALFF)* [7] is a frequency domain metric representing the relative contribution of specific oscillations to the entire frequency range. It is based on the ratio of the amplitudes of fluctuations in the 0.01-0.1 Hz frequency range to the sum of amplitudes in the entire frequency spectrum. No temporal filtering was applied, because the data was analyzed in the frequency domain. Fractional ALFF maps at the subject-level were transformed into subject-level *Z*-score maps.

**Conjunction analyses**

Spatial overlap was obtained by conjunction analyses of the statistical *Z*-maps with logical ‘AND’ masking [11]. The extent of overlap for each conjunction contrast was quantified as the average of the proportion of the total number of suprathreshold voxels for each map [12-14]. To illustrate, if *A* is a contrast-map stemming from the ASD-related differences sample and *B* is a contrast-map stemming from the typical sex differences sample, the following procedure will be applied:

1. C = (A AND B)/A
2. D = (A AND B)/B
3. Overlap percentage = ((C + D)/2)*100

**Significance testing**

To test for statistical significance of the spatial overlap we ran 5000 Monte Carlo simulations (MCS) across 500 voxel-level thresholds for each derivative to generate the null distribution of random spatial overlap. To match the distribution of *Z*-values in the brain, MCS are an ideal choice as they are based on values randomly sampled from a Gaussian distribution.

In supplementary post-hoc analyses we verified the robustness of findings based on MCS using permutation testing. Specifically, we ran 1000 permutations at the voxel-level threshold *Z* ≥ 2.58 by randomly resampling group-memberships for each statistical group comparison (i.e., ASD vs. NT using ABIDE I and NT M vs. NT F using FCP) emerging from the analyses conducted after aligning pre-processing methods. We calculated the percentage of overlap at the 99.5^th^ percentile of the null distribution of 1000 random overlaps (originating from the contrasts based on spatially permuted group labels). This was then compared to the real overlap at *Z* ≥ 2.58. We only ran 1000 permutations given that they are computationally costly and systematic methods comparisons are beyond the scope of the study. Nevertheless, results of the above permutations were consistent with the main findings as summarized in Additional file 6: Table S2.

**Individual and group analyses for strategies 2-4**

The specific processing steps taken to generate the original *Z*-maps are described elsewhere [1, 2]; below we detail the preprocessing analyses applied to generate the *Z*-maps used for conjunction analyses in Strategies 2 to 4.

Data were analyzed using version 0.3.9.1 of the Configurable Pipeline for the Analysis of Connectomes [15] (C-PAC, http://fcp-indi.github.com/C-PAC/), which integrates tools from AFNI (<http://afni.nimh.nih.gov/afni>), FSL (<http://fmrib.ox.ac.uk>) and Advanced Normalization Tools (ANTs; <http://stnava.github.io/ANTs>) using Nipype (<http://nipype.readthedocs.io/en/latest/>).

***Structural preprocessing***

1. Skull-stripping: T1-weighted images were skull-stripped using FSL’s *BET* command.
2. Tissue Segmentation: FSL’s FAST command was used to segment images into GM, white matter (WM) and cerebrospinal fluid (CSF). Probability thresholds were 0.96 for WM and CSF and 0.7 for GM.
3. Spatial normalization: images (with skull-on) were normalized to MNI152 stereotactic space (2mm isotropic) with linear and non-linear registrations using ANTs. For the calculation of VMHC, spatial normalization was done by registering to a symmetrical template.

***Functional preprocessing***

1. Slice time correction: the AFNI command *3dTshift* was used to correct for differences in acquisition time between the slices using the specific parameters for each site based on their acquisition protocols (ABIDE I) or information provided online (FCP and GSP).
2. Motion realignment: motion correction was performed using the AFNI command *3dvolreg* by a two-pass procedure. In a first step, each functional volume was co-registered to the (un-aligned) mean functional image. In a second step, a new functional mean image based on the aligned images was used as the reference image. At this second stage, motion parameters based on the Friston 24-Parameter Model (six motion parameters, their values of preceding volumes, 12 squared values of these items) were calculated along with mean framewise displacement (mFD; based on Jenkinson et al. [16]).
3. Skull-stripping: skull was removed using the AFNI command *3dAutomask*.
4. Mean-based intensity normalization: all images were scaled with a factor of 10.000.
5. Nuisance signal regression: 24 motion parameters based on Friston 24-Parameter Model [17] were regressed out, along with mean signals from WM and CSF obtained using subject-specific masks with tissue probability thresholds of 0.96 for WM and CSF. Linear and quadratic trends were additionally included as nuisance regressors.
6. Temporal filtering: band-pass filtering (0.01-0.1 Hz) was done using the AFNI command *3dBandpass*. This was done for all R-fMRI derivatives other than fALFF.
7. Registration: functional-to-anatomical co-registration was achieved by Boundary Based Registration (BBR) using FSL FLIRT. Spatial normalization of functional EPIs to MNI152 space was done by applying linear and non-linear transforms from ANTs.
8. SCA analyses were carried out using a PCC seed centered at MNI coordinates x=0, y=-53, z=26 [10] with 8mm diameter sphere. ReHo, fALFF, and SCA were calculated in native space, before being transformed into MNI152 space. DC was calculated in MNI152 space. VMHC was calculated based on smoothed data in symmetric MNI152 space.
9. Spatial filtering: Derivatives (fALFF, ReHo, SCA, DC) were smoothed with a 3D Gaussian kernel (Full-Width-Half-Maximum [FWHM]=6mm) after computing and registering each derivative. VMHC was spatially filtered (FWHM=6mm) prior to its calculation and registration.

Due to registration failure, four individual data in the FCP sample (1 male from Beijing, 2 females from Oulu, 1 female from NYU) and four individual data subjects in the ABIDE I sample (2 ASD from UM 1, 1 ASD from UM 2, 1 ASD from Yale) were excluded, resulting in 824 FCP data comprising 356 NT males and 468 NT females, and 759 ABIDE data comprising 356 individuals with ASD and 403 NT controls.

Statistical individual *Z*-maps were generated within study-specific functional volume masks including voxels (in MNI space) present across all individual data in a given study/sample (i.e., FCP and ABIDE I, separately). A general linear model was fitted at each voxel including group (i.e., diagnosis [ASD–NT] for the ABIDE I sample and sex [NT M–NT F] for the FCP sample), age, site, mFD [16] and individual subject means of each R-fMRI derivative as regressors. The patterns of results emerging from group comparisons were similar across strategies.

**Samples age matching of analysis strategy 3**

As shown in Additional file 4: Fig. S2, age matching was performed at the background of following criteria: (1) since NYU donated de-identified data in both ABIDE I and FCP, we first examined if they were likely from the same individuals. We correlated the T1 image of each individual ID in one of the two NYU collections, with all other T1s in the other one. This was performed on data registered in MNI space, masked to only include the voxels shared across all NYU individuals. Six ID pairs yielded a nearly perfect correspondence (i.e. *r*=0.98-1). Among these, we only retained the six ID from ABIDE I (due to smaller sample size of ABIDE I compared to FCP); (2) to ensure a balanced representation of sites we only retained sites with at least eight individuals per group (i.e., ASD and NT in ABIDE I and males and females in FCP). This resulted in the exclusion of eight data collections in FCP; (3) given that FCP included only 19 individuals under 17 years of age, we narrowed the selection to those data of individuals aged 17.5 to 37 years. This was based on the lower and upper 5^th^ percentiles of the FCP age range (after above exclusions). After excluding individuals beyond the newly defined age range, sites that did no longer meet criterion (2) were additionally excluded – these were five sites in ABIDE I and two sites in FCP. The resulting sample was then statistically matched for age by gradually excluding up to 61 additional males and up to 120 additional females from the FCP sample from the sites with largest numbers of individuals (Beijing, Cambridge and Oulu). As a result, there were no significant differences in age between males with ASD and NT controls (*t_197_* = -0.206; *p* = 0.837), between NT males and NT females (*t_435_* = 0.225; *p* = 0.822), as well as between the ABIDE I and FCP cohorts (*t_278_* = 1.645; *p* = 0.101). For details on sample characteristics see Additional file 5: Table S1.

**Selection of the Brain Genomics Superstruct dataset (analysis strategy 4)**

Using the same criteria as specified above for Strategy 3, 742 data from the GSP repository were selected to age match both the ABIDE I and FCP subsamples used for Strategy 3. As a result, this GSP subsample included 320 males and 422 females aged 18 to 35 years. There were no significant differences in age between GSP males and females (*t_437_* = 1.073; *p* = 0.284), between the GSP and FCP age-matched cohorts (*t_1179_* = 0.407; *p* = 0.684), and between the GSP and ABIDE I age-matched cohorts (*t_241_* = -1.51; *p* = 0.135). Details on GSP data acquisition can be found on the GSP website (<http://neuroinformatics.harvard.edu/gsp/>). For details on sample characteristics see Additional file 5: Table S1.

**Characterization of conjunction maps**

To characterize results we computed the overlap of conjunction maps thresholded at *Z* ≥ 2.58 with the seven functional cortical networks described by Yeo et al. [18] and 12 cognitive ontology maps by Yeo et al. [19]. In each case we then established the percentage of voxels within each conjunction result that was part of the Yeo maps.

To establish how much percentage of ASD-related atypicalities were driven by an overlap with typical sex differences (regardless of the EMB or GI model predictions), we calculated the percentage of voxels within each conjunction map that was part of the ASD-related difference maps across all 500 voxel-level thresholds as described in the main methods. The inter-quartile range across thresholds was calculated for each R-fMRI derivative separately.

**Similarity across analytical strategies 1-4**

In order to characterize the similarity of results across the four different analytical strategies, we computed all possible overlap percentages between each one of the four strategies and the remaining three strategies across all 500 voxel-level thresholds (Additional files 13 and 14: Tables S5 and S6). We calculated the significant overlap percentage for each conjunction analysis [13, 14] using the same formula as above.

**References**

1. Di Martino A, Yan CG, Li Q, Denio E, Castellanos FX, Alaerts K, Anderson JS, Assaf M, Bookheimer SY, Dapretto M, et al: **The autism brain imaging data exchange: towards a large-scale evaluation of the intrinsic brain architecture in autism.** *Mol Psychiatry* 2014, **19:**659-667.

2. Yan CG, Craddock RC, Zuo XN, Zang YF, Milham MP: **Standardizing the intrinsic brain: towards robust measurement of inter-individual variation in 1000 functional connectomes.** *Neuroimage* 2013, **80:**246-262.

3. Holmes AJ, Hollinshead MO, O'Keefe TM, Petrov VI, Fariello GR, Wald LL, Fischl B, Rosen BR, Mair RW, Roffman JL, et al: **Brain Genomics Superstruct Project initial data release with structural, functional, and behavioral measures.** *Sci Data* 2015, **2:**150031.

4. Zang Y, Jiang T, Lu Y, He Y, Tian L: **Regional homogeneity approach to fMRI data analysis.** *Neuroimage* 2004, **22:**394-400.

5. Zuo XN, Kelly C, Di Martino A, Mennes M, Margulies DS, Bangaru S, Grzadzinski R, Evans AC, Zang YF, Castellanos FX, Milham MP: **Growing together and growing apart: regional and sex differences in the lifespan developmental trajectories of functional homotopy.** *J Neurosci* 2010, **30:**15034-15043.

6. Zuo XN, Ehmke R, Mennes M, Imperati D, Castellanos FX, Sporns O, Milham MP: **Network centrality in the human functional connectome.** *Cereb Cortex* 2012, **22:**1862-1875.

7. Zou QH, Zhu CZ, Yang Y, Zuo XN, Long XY, Cao QJ, Wang YF, Zang YF: **An improved approach to detection of amplitude of low-frequency fluctuation (ALFF) for resting-state fMRI: fractional ALFF.** *J Neurosci Methods* 2008, **172:**137-141.

8. Kendall MG, Gibbons JD: *Rank correlation methods.* 5th ed. edn: Edward Arnold; 1990.

9. Satterthwaite TD, Wolf DH, Loughead J, Ruparel K, Elliott MA, Hakonarson H, Gur RC, Gur RE: **Impact of in-scanner head motion on multiple measures of functional connectivity: relevance for studies of neurodevelopment in youth.** *Neuroimage* 2012, **60:**623-632.

10. Andrews-Hanna JR, Snyder AZ, Vincent JL, Lustig C, Head D, Raichle ME, Buckner RL: **Disruption of large-scale brain systems in advanced aging.** *Neuron* 2007, **56:**924-935.

11. Nichols T, Brett M, Andersson J, Wager T, Poline JB: **Valid conjunction inference with the minimum statistic.** *Neuroimage* 2005, **25:**653-660.

12. Holt RJ, Chura LR, Lai MC, Suckling J, von dem Hagen E, Calder AJ, Bullmore ET, Baron-Cohen S, Spencer MD: **'Reading the Mind in the Eyes': an fMRI study of adolescents with autism and their siblings.** *Psychol Med* 2014, **44:**3215-3227.

13. Lai MC, Lombardo MV, Ecker C, Chakrabarti B, Suckling J, Bullmore ET, Happé F, Murphy DG, Baron-Cohen S, Consortium MA: **Neuroanatomy of Individual Differences in Language in Adult Males with Autism.** *Cereb Cortex* 2014.

14. Lai MC, Lombardo MV, Suckling J, Ruigrok AN, Chakrabarti B, Ecker C, Deoni SC, Craig MC, Murphy DG, Bullmore ET, et al: **Biological sex affects the neurobiology of autism.** *Brain* 2013, **136:**2799-2815.

15. Craddock RC, Sikka S, Cheung B, Khanuja R, Ghosh SS, Yan C, Li Q, Lurie D, Vogelstein j, Burns R, et al: **Towards Automated Analysis of Connectomes: The Configurable Pipeline for the Analysis of Connectomes (C-PAC).** In *Neuroinformatics* Stockholm, Sweden: Frontiers; 2013.

16. Jenkinson M, Bannister P, Brady M, Smith S: **Improved optimization for the robust and accurate linear registration and motion correction of brain images.** *Neuroimage* 2002, **17:**825-841.

17. Friston KJ, Williams S, Howard R, Frackowiak RS, Turner R: **Movement-related effects in fMRI time-series.** *Magn Reson Med* 1996, **35:**346-355.

18. Yeo BT, Krienen FM, Sepulcre J, Sabuncu MR, Lashkari D, Hollinshead M, Roffman JL, Smoller JW, Zöllei L, Polimeni JR, et al: **The organization of the human cerebral cortex estimated by intrinsic functional connectivity.** *J Neurophysiol* 2011, **106:**1125-1165.

19. Yeo BT, Krienen FM, Eickhoff SB, Yaakub SN, Fox PT, Buckner RL, Asplund CL, Chee MW: **Functional Specialization and Flexibility in Human Association Cortex.** *Cereb Cortex* 2015, **25:**3654-3672.
