# Supplementary material for: Network-specific sex differentiation of intrinsic brain function in males with autism
Source: Mol Autism. 2018 Mar 6;9:17. doi: 10.1186/s13229-018-0192-x (PMC5840786; doi:10.1186/s13229-018-0192-x)
Supplement: Supplementary file 3 — Supplementary results. (DOCX 120 kb) [file 13229_2018_192_MOESM3_ESM.docx]

**Additional file 3: Supplementary results**

Secondary analyses aligning image processing analyses without (Strategy 2) or with alignment for sample age (Strategy 3) and using an independent neurotypical (NT) sample (Strategy 4), all yielded a pattern of findings similar to our primary analysis (Strategy 1).

**Results consistent with a shift-towards-maleness (EMB)**

As shown in Additional file 9: Fig. S3, results of conjunction analyses of *Z*-maps resulting from different strategies were consistent with a shift-towards-maleness (EMB) across all R-fMRI metrics but DC, as also resulting form our primary analyses (Strategy 1; Figure 2). As summarized in Additional file 14: Table S6, consistency across strategies was evident in regard to neural network involved by the EMB scenarios (fronto-parietal [FP] for EMB 1 and default network [DN] for EMB 2). Overall, EMB 2 showed the highest consistency across differently applied strategies (30.4%) (Additional file 14: Table S6). As also seen in primary analyses, a shift-towards-maleness ASD-related hyper-connectivity (EMB 1) in ReHo predominantly encompassed the FP network and to a lesser extent, the ventral attention (VA) network and DN (Additional file 10: Fig. S4). Only Strategy 4 showed ASD-related increases also in fALFF in these same networks. As shown in Additional file 10: Fig. S4, ASD-related decreases (EMB 2) in local (ReHo), homotopic (VMHC) and PCC-iFC, as well as in fALFF, predominantly encompassed the DN mainly centered around precuneus and PCC. This pattern showed the highest consistency for PCC-iFC (44.7%) and VMHC (43.9%), followed by ReHo (36.31%), whereas fALFF was the least consistent (18.99%) (Additional file 14: Table S6).

**Results consistent with a shift-towards-femaleness (GI)**

Consistency across analytical strategies was also observed in regard to the neural networks involved by GI scenarios – i.e., somatomotor (SM) network for GI 2 and DN for GI 1. However, there was more variation across strategies in terms of which R-fMRI metrics expressed these patterns. While overlaps consistent with a shift-towards-femaleness characterized ReHo, VMHC and DC across all strategies, findings for PCC-iFC and fALFF varied by analytical strategy. Both GI 1 and GI 2 showed on average moderately consistent values across different strategies (15.1% and 17.07%, respectively; see Additional file 14: Table S6).

As shown in Additional file 11: Fig. S5, ASD-related decreases (GI 2) in local (ReHo) and homotopic (VMHC) connectivity, and network centrality (DC) predominantly encompassed the SM network and to a lesser degree, the visual (VS), VA and dorsal attention (DA) networks. This was additionally expressed in Strategy 2 in the PCC-iFC and in Strategy 4 in fALFF, however with greater involvements of the VS network. This pattern showed highest consistency across VMHC and ReHo, followed by DC and PCC-iFC (see Additional file 14: Table S6). ASD-related hyper-connectivity in ASD (GI 1) mainly encompassed the DN across ReHo and DC across all four strategies (see Additional file 11: Fig. S5). This was additionally present in Strategy 4 in fALFF, however not in Strategy 2 in VMHC. This pattern showed highest consistency across ReHo, followed by DC, VMHC and PCC-iFC (see Additional file 14: Table S6).

**Cognitive ontology in secondary analyses**

To explore the cognitive domains implicated in the EMB and GI clusters described above, we quantified the percentage of their overlap with the 12-cognitive ontology maps by Yeo et al. [1] (thresholded at *p* = 1e-5). Across ReHo, VMHC, fALFF and PCC-iFC, probability maps activated by social tasks such as theory of mind, face emotion processing and higher-order visual processing strongly overlapped with EMB 2 findings (Additional file 12: Fig. S6). In contrast, the highest overlap for ASD-related, increases consistent with a shift-towards-maleness in ReHo (EMB 1) occurred with the cognitive components recruited by non-social cognitive control tasks (see Additional file 12: Fig. S6).

The most strongly implicated cognitive components across DC, ReHo, VMHC and PCC-iFC consistent with the GI 2 model included motor tasks related to hand, oral and ocular movement (Additional file 12: Fig. S6). In contrast, the highest overlap for ASD-related increases consistent with a shift-towards-femaleness in ReHo and DC (GI 1) occurred with cognitive components recruited by executive control.

**Supplementary Figure Legends**

**Additional File 1: Figure S1. ABIDE and FCP results.**

For each of the five resting-state fMRI (R-fMRI) metrics examined, the left panel shows significant group differences between males with ASD and neurotypical males (NT M) based on the original Autism Brain Imaging Data Exchange (ABIDE) study [2]; the right panel shows group differences between NT M and NT females (F) based on the original 1000 Functional Connectome Project (FCP) study [3]. Gaussian random filed theory was employed to apply cluster-level corrections for multiple comparisons, with a statistical voxel-level threshold of *Z* ≥ 2.58 and cluster-level significance of *p* < 0.05. R-fMRI abbreviations: DC = degree centrality; fALFF = fractional amplitude of low frequency fluctuations; ReHo = regional homogeneity; VMHC = voxel-mirrored homotopic connectivity; PCC-iFC = posterior cingulate cortex intrinsic functional connectivity.

**Additional File 4: Figure S2. Age Matching.**

Age-matching was done according to the following criteria: 1) exclude individuals common to FCP and ABIDE I; 2) exclude data from sites with less than eight individuals per site per group; 3) exclude data from individuals with age outside the lower and upper 5^th^ percentile of the FCP age distribution; 4) exclude any data collection with less than eight datasets after the above steps. The boxes on each side provide information on the site and data excluded for ABIDE I (left) and FCP sample (right).

**Additional file 9: Figure S3. Conjunction Analyses Across Strategies 2-4.**

For each analytical strategy, plots (one per resting-state fMRI [R-fMRI] metric examined) show the spatial overlap percentages across 500 voxel-level statistical thresholds for the four overlap scenarios (turquoise: shift-towards-maleness (STM) ASD-related increases (EMB 1) = ASD♂>NT♂ & NT♂>NT♀; blue: STM ASD-related decreases (EMB 2) = ASD♂<NT♂ & NT♂<NT♀; orange: shift-towards-femaleness (STF) ASD-related increases (GI 1) = ASD♂>NT♂ & NT♂<NT♀; yellow: STF ASD-related decreases (GI 2) = ASD♂<NT♂ & NT♂>NT♀). The black solid line represents the median of null distribution of the random spatial overlap generated by 5000 Monte Carlo simulations for each threshold; the dotted lines mark the 0.5^st^ and 99.5^th^ percentiles of the null distribution of random spatial overlap. Strategy 2 = conjunction of *Z*-maps obtained with a common analytical pipeline in ABIDE I and FCP samples; Strategy 3 = conjunction of *Z*-maps obtained with age-matched subsamples and a common pipeline; Strategy 4 = replication with an independent sex difference sample (GSP) using age-matched subsamples and a common pipeline as above. R-fMRI abbreviations: DC = degree centrality; fALFF = fractional amplitude of low frequency fluctuations; ReHo = regional homogeneity; VMHC = voxel-mirrored homotopic connectivity; PCC-iFC = posterior cingulate cortex intrinsic functional connectivity.

**Additional File 10: Figure S4. Overlaps Consistent with a Shift-Towards-Maleness (EMB) across Strategies 2–4.**

For each analytical strategy column and for each resting-state fMRI (R-fMRI) metric examined, inflated surface maps show the regions of significant spatial overlap based on the conjunction of statistical *Z*-maps voxel-level thresholded at *Z* ≥ 2.58. The histograms in the right column describe the percentage of voxels within the above clusters included in the seven functional cortical networks described by Yeo et al. [4]. Strategy 2 = conjunction of *Z*-maps obtained with a common analytical pipeline in ABIDE I and FCP samples; Strategy 3 = analyses conducted with age-matched subsamples and a common pipeline; Strategy 4 = replication with an independent sex difference sample (GSP) using age-matched subsamples and a common pipeline as above. Color codes: turquoise = shift-towards-maleness (STM) ASD-related increases (EMB 1); blue = STM ASD-related decreases (EMB 2); R-fMRI abbreviations: DC = Degree Centrality; fALFF = fractional amplitude of low frequency fluctuations; ReHo = regional homogeneity; VMHC = voxel-mirrored homotopic connectivity; PCC-iFC = posterior cingulate cortex intrinsic functional connectivity. Seven functional Yeo networks: VS = visual network; SM = somatomotor network; DA = dorsal attention network; VA = ventral attention network; LB = limbic network; FP = fronto-parietal network; DN = default network.

**Additional File 11: Figure S5. Overlaps Consistent with a Shift-Towards-Femaleness (GI) across Strategies 2–4.**

For each analytical strategy column and for each resting-state fMRI (R-fMRI) metric examined surface maps show the regions of significant spatial overlap based on the conjunction of statistical *Z*-maps voxel-level thresholded at *Z* ≥ 2.58 across the three secondary analyses. The histograms in the right column describe the percentage of voxels within the above clusters included in the seven functional cortical networks described by Yeo et al. [4]. Strategy 2 = conjunction of *Z*-maps obtained with a common analytical pipeline in ABIDE I and FCP samples; Strategy 3 = analyses conducted with age matched subsamples and a common pipeline; Strategy 4 = replication with an independent sex difference sample (GSP) using age-matched subsamples and a common pipeline as above. Color codes: orange = shift-towards-femaleness (STF) ASD-related increases (GI 1); yellow = STF ASD-related decreases (GI 2). R-fMRI abbreviations: DC = degree centrality; fALFF = fractional amplitude of low frequency fluctuations; ReHo = regional homogeneity; VMHC = voxel-mirrored homotopic connectivity; PCC-iFC = posterior cingulate cortex intrinsic functional connectivity. Seven functional Yeo networks: VS = visual network; SM = somatomotor network; DA = dorsal attention network; VA = ventral attention network; LB = limbic network; FP = fronto-parietal network; DN = default network.

**Additional File 12: Figure S6. Cognitive Ontology Maps for Strategies 2-4.**

The polar plots show the percentage (0-80%) of overlap between the significant conjunctions of statistical *Z*-maps (voxel-level thresholded at *Z* ≥ 2.58) and the 12 Yeo cognitive ontology probability maps [1] for cognitive components C1-C12 (probability thresholded at *p* = 1e-5) for Strategies 2 to 4. Strategy 2 = conjunction of *Z*-maps obtained with a common analytical pipeline in ABIDE I and FCP samples; Strategy 3 = analyses conducted with age matched subsamples and a common pipeline; Strategy 4 = replication with an independent sex difference sample (GSP) using age-matched subsamples and a common pipeline as above. Results are summarized according to their consistency with the two models, regardless of the resting-state fMRI metric. Color codes: turquoise = shift-towards-maleness (STM) ASD-related increases (EMB 1); blue = STM ASD-related decreases (EMB 2); orange = shift-towards-femaleness (STF) ASD-related increases (GI 1); yellow = STF ASD-related decreases (GI 2).

**References**

1. Yeo BT, Krienen FM, Eickhoff SB, Yaakub SN, Fox PT, Buckner RL, Asplund CL, Chee MW: **Functional Specialization and Flexibility in Human Association Cortex.** *Cereb Cortex* 2015, **25:**3654-3672.

2. Di Martino A, Yan CG, Li Q, Denio E, Castellanos FX, Alaerts K, Anderson JS, Assaf M, Bookheimer SY, Dapretto M, et al: **The autism brain imaging data exchange: towards a large-scale evaluation of the intrinsic brain architecture in autism.** *Mol Psychiatry* 2014, **19:**659-667.

3. Yan CG, Craddock RC, Zuo XN, Zang YF, Milham MP: **Standardizing the intrinsic brain: towards robust measurement of inter-individual variation in 1000 functional connectomes.** *Neuroimage* 2013, **80:**246-262.

4. Yeo BT, Krienen FM, Sepulcre J, Sabuncu MR, Lashkari D, Hollinshead M, Roffman JL, Smoller JW, Zöllei L, Polimeni JR, et al: **The organization of the human cerebral cortex estimated by intrinsic functional connectivity.** *J Neurophysiol* 2011, **106:**1125-1165.
