# Supplementary material for: Network-specific sex differentiation of intrinsic brain function in males with autism
Source: Mol Autism. 2018 Mar 6;9:17. doi: 10.1186/s13229-018-0192-x (PMC5840786; doi:10.1186/s13229-018-0192-x)
Supplement: Supplementary file 5 — Characterization of age-matched ABIDE I, FCP and GSP samples. (DOCX 27 kb) [file 13229_2018_192_MOESM5_ESM.docx]

**Additional File 5: Table S1. Characterization of age-matched ABIDE I, FCP and GSP samples.**

| **ABIDE I** | **Sites^a^** | **ASD (n=93)** | **NT (n=106)** | **Statistics** |
| --- | --- | --- | --- | --- |
|  | **N** | **N/Mean (SD) [Range]** | **N/Mean (SD) [Range]** |  |
| Sex | 6 | 93 males | 106 males | - |
| Age | 6 | 23.9 (5.2) [17.7 – 35.7] | 24 (4.4) [17.5 – 35] | *t_197_*=-0.2, *p*=.8 |
| Full-Scale IQ^b^ | 6 | 107.6 (14.6) [78 – 137] | 113.7 (10.2) [89 – 139] | *t_159_*=-3.4, *p*=.001 |
| Verbal IQ^c^ | 5 | 105.7 (16.6) [69 – 136] | 113.6 (11) [88 – 140] | *t_144_*=-3.6, *p*<.001 |
| Performance IQ^d^ | 6 | 108.1 (15) [72 – 149] | 111.1 (10.9) [83 – 132] | *t_155_*=-1.6, *p*=.1 |
| ADI-R |  |  |  |  |
| Social^e^ | 3 | 20.7 (5.3) [9 – 30] | - | - |
| Communication^e^ | 3 | 17.1 (4.7) [8 – 26] | - | - |
| RRB^e^ | 3 | 5.8 (3) [2 – 12] | - | - |
| ADOS-G (Lord et al.) |  |  |  |  |
| Communication^f^ | 3 | 4.4 (1.2) [2 – 7] | - | - |
| Social^g^ | 3 | 7.16 (3.8) [0 – 14] | - | - |
| RRB^h^ | 3 | 1.68 (1.55) [0 – 8] | - | - |
| Comorbidity | 1 | 1 (NYU)^i^ | - | - |
| **FCP** | **Sites^j^** | **Males (n=183)** | **Females (n=256)** | **Statistics** |
|  | **N** | **N/Mean (SD) [Range]** | **N/Mean (SD) [Range]** |  |
| Sex | 8 | 183 males | 256 females | - |
| Age | 8 | 23.4 (3.42) [18 – 37] | 23.34 (2.94) [18 – 36] | *t_437_*=0.2, *p*=.8 |
| **GSP** | **Sites^k^** | **Males (n=320)** | **Females (n=422)** | **Statistics** |
|  | **N** | **N/Mean (SD) [Range]** | **N/Mean (SD) [Range]** |  |
| Sex | 4 | 320 males | 422 females | - |
| Age^l^ | 4 | 23.57 (3.14) [18 - 35] | 23.34 (2.89) [18 – 35] | *t_740_*=-1.1, *p*=.3 |

^a^ Included sites were Leuven 1, Max Mun, NYU, Pitt, Trinity and USM. ^b^ Information was available for 92 individuals with ASD and 106 neurotypical controls (NT). ^c^ Information was available for 84 individuals with ASD and 85 NT. ^d^ Information was available for 87 individuals with ASD and 100 NT. ^e^ Information was available for 32 individuals. ^f^ Information was available for 60 individuals with ASD. ^g^ Information was available for 69 individuals with ASD. ^h^ Information was available for 57 individuals with ASD. ^i^Attention Deficit Hyperactivity disorder (ADHD; N=1). ^j^ Included sites were Baltimore, Beijing, Cambridge, Leiden 2, Leipzig, New York 1, Oulu and Saint Louis. ^k^ One site was excluded due to less than 8 individuals per group. ^l^ Age was available in binned form (2 year bins). Participants who were from 18-19 years of age at the point of scan are coded as 19, participants who were 20-21 years of age are coded as 21, etc. Mean and SD are based on these age bins.
