# Supplementary material for: Network-specific sex differentiation of intrinsic brain function in males with autism
Source: Mol Autism. 2018 Mar 6;9:17. doi: 10.1186/s13229-018-0192-x (PMC5840786; doi:10.1186/s13229-018-0192-x)
Supplement: Supplementary file 6 — Comparison of percentage of overlaps at Z ≥ 2.58 for the real overlap, random overlap generated by 5000 Monte Carlo simulation, and random overlap generated by 1000 permutations, across R-fMRI metrics by model. (DOCX 147 kb) [file 13229_2018_192_MOESM6_ESM.docx]

| **Strategy 2** | **DC** | | | **fALFF** | | | **ReHo** | | | **VMHC** | | | **PCC-iFC** | | |
| --- | --- | --- | --- | --- | --- | --- | --- | --- | --- | --- | --- | --- | --- | --- | --- |
| **Models** | PM | MC | RO | PM | MC | RO | PM | MC | RO | PM | MC | RO | PM | MC | RO |
| **EMB 1 (STM** ↑**)** | 3.34 | 8.22 | 0.66 | 3.06 | 4.7 | 1.4 | 2.82 | 4.04 | 4.14** | 3.72 | 2.76 | 0.24 | 2.75 | 4.03 | 0.11 |
| **EMB 2 (STM** ↓**)** | 2.28 | 8.22 | 0.41 | 2.24 | 4.7 | 10.04** | 4.98 | 4.04 | 9.43** | 3.53 | 2.76 | 14.69** | 3.51 | 4.03 | 12.69** |
| **GI 1 (STF** ↑**)** | 2.56 | 8.22 | 9.36** | 2.6 | 4.7 | 1.77 | 3.44 | 4.04 | 8.14** | 4.27 | 2.76 | 0.73 | 4.47 | 4.03 | 2.09 |
| **GI 2 (STF** ↓**)** | 2.6 | 8.22 | 9.79** | 2.37 | 4.7 | 1.02 | 3.4 | 4.04 | 2.4 | 4.27 | 2.76 | 3.78* | 4.25 | 4.03 | 2.38 |

**Additional File 6: Table S2. Comparison of percentage of overlaps at *Z* ≥ 2.58 for the real overlap, random overlap generated by 5000 Monte Carlo simulations, and random overlap generated by 1000 permutations, across R-fMRI metrics by model**

Significance of overlap is determined by whether the real overlap is larger than the 99.5^th^ percentile of randomly generated null distributions of overlaps with either the Monte Carlo simulations (as per our main findings) or a supplementary permutation testing, at the voxel-level threshold *Z* ≥ 2.58. One* shows where the real overlap percentage is larger than the 99.5^th^ percentile of the random overlap percentage derived from Monte Carlo simulations *or* permutation testing. Two** show where the real overlap percentage is larger than the 99.5^th^ percentile of the random overlap percentage derived from Monte Carlo simulations *and* permutation testing. Abbreviations: PM = percentage of random overlap from 1000 permutations at *Z* ≥ 2.58; MC = percentage of random overlap from 5000 Monte Carlo simulations at *Z* ≥ 2.58; RO = percentage of real overlap; Strategy 2 = conjunction of *Z*-maps obtained with a common analytical pipeline in ABIDE I and FCP samples; DC = degree centrality; fALFF = fractional amplitude of low frequency fluctuations; PCC-iFC = posterior cingulate cortex intrinsic functional connectivity; ReHo = regional homogeneity; VMHC = voxel-mirrored homotopic connectivity; STM = shift-towards-maleness; STF = shift-towards-femaleness; turquoise: EMB 1 = ASD♂>NT♂ & NT♂>NT♀; blue: EMB 2 = ASD♂<NT♂ & NT♂<NT♀; orange: GI 1 = ASD♂>NT♂ & NT♂<NT♀; yellow: GI 2 = ASD♂<NT♂ & NT♂>NT♀.
