# Supplementary material for: Network-specific sex differentiation of intrinsic brain function in males with autism
Source: Mol Autism. 2018 Mar 6;9:17. doi: 10.1186/s13229-018-0192-x (PMC5840786; doi:10.1186/s13229-018-0192-x)
Supplement: Supplementary file 7 — Percentages of voxels within the conjunction maps (thresholded at Z ≥ 2.58) overlapping with the seven functional cortical networks per Yeo et al. [40]. (DOCX 139 kb) [file 13229_2018_192_MOESM7_ESM.docx]

**Additional File 7: Table S3. Percentages of voxels within the conjunction maps (thresholded at *Z*** ≥ **2.58) overlapping with the seven functional cortical networks per Yeo *et al.* [40]**

|  | **VS** | **SM** | **DA** | **VA** | **LB** | **FP** | **DN** |
| --- | --- | --- | --- | --- | --- | --- | --- |
| **EMB 1 (STM** ↑**)** |  |  |  |  |  |  |  |
| ReHo | 1.75% | 1.75% | 1.75% | 19.3% | - | 57.9% | 14.62% |
| **EMB 2 (STM** ↓**)** |  |  |  |  |  |  |  |
| fALFF | - | - | 3.23% | - | - | - | 38.71% |
| ReHo | 5.77% | 0.64% | - | - | - | 1.28% | 73.72% |
| VMHC | 1.57% | - | 0.45% | 3.36% | - | 2.24% | 47.31% |
| PCC-iFC | 0.4% | - | 0.4% | - | - | 7.65% | 80.89% |
| **GI 1 (STF** ↑**)** |  |  |  |  |  |  |  |
| DC | - | - | 0.5% | 0.5% | 0.5% | 3.92% | 16.67% |
| ReHo | - | - | 5.51% | 3.86% | - | 25.07% | 24.8% |
| **GI 2 (STF** ↓**)** |  |  |  |  |  |  |  |
| DC | 2.56% | 66.67% | 11.54% | 2.56% | - | - | - |
| ReHo | 5.74% | 43.44% | - | 42.62% | - | - | - |
| VMHC | 5.28% | 67.74% | 4.99% | 2.93% | - | 1.17% | 8.21% |
| PCC-iFC | 2.94% | 61.76% | 1.47% | 25% | - | - | - |

VS = visual network; SM = somatomotor network; DA = dorsal attention network; VA = ventral attention network; LB = limbic network; FP = fronto-parietal network; DN = default network; DC = degree centrality; fALFF = fractional amplitude of low frequency fluctuations; PCC-iFC = posterior cingulate cortex intrinsic functional connectivity; ReHo = regional homogeneity; VMHC = voxel-mirrored homotopic connectivity; turquoise: EMB 1 = ASD♂>NT♂ & NT♂>NT♀; blue: EMB 2 = ASD♂<NT♂ & NT♂<NT♀; orange: GI 1 = ASD♂>NT♂ & NT♂<NT♀; yellow: GI 2 = ASD♂<NT♂ & NT♂>NT♀.
