# Supplementary material for: Network-specific sex differentiation of intrinsic brain function in males with autism
Source: Mol Autism. 2018 Mar 6;9:17. doi: 10.1186/s13229-018-0192-x (PMC5840786; doi:10.1186/s13229-018-0192-x)
Supplement: Supplementary file 8 — Percentage of voxels within the conjunction maps (thresholded at Z ≥ 2.58) and the 12 cognitive ontology maps defined Yeo et al. [41] (probability thresholded at p = 1e-5). (DOCX 178 kb) [file 13229_2018_192_MOESM8_ESM.docx]

**Additional File 8: Table S4. Percentage of voxels within the conjunction maps (thresholded at *Z*** ≥ **2.58) and the 12 cognitive ontology maps defined Yeo *et al.* [41] (probability thresholded at *p* = 1e-5)**

|  | **C1** | **C2** | **C3** | **C4** | **C5** | **C6** | **C7** | **C8** | **C9** | **C10** | **C11** | **C12** |
| --- | --- | --- | --- | --- | --- | --- | --- | --- | --- | --- | --- | --- |
| **EMB 1 (STM** ↑**)** |  |  |  |  |  |  |  |  |  |  |  |  |
| ReHo | - | 0.58% | 2.34% | - | - | 11.11% | 1.75% | 66.67% | 56.14% | 9.36% | - | 1.75% |
| **EMB 2 (STM** ↓**)** |  |  |  |  |  |  |  |  |  |  |  |  |
| fALFF | - | - | - | 3.23% | - | - | - | 9.68% | 35.48% | 77.42% | 16.13% | 19.35% |
| ReHo | 1.92% | 6.41% | 6.41% | 4.49% | 12.82% | - | 8.97% | 10.26% | 16.03% | 77.56% | 42.95% | 15.38% |
| VMHC | 15.47% | 14.8% | 8.52% | 2.47% | 6.95% | 0.22% | 23.32% | 37% | 12.56% | 42.83% | 21.97% | 39.69% |
| PCC-iFC | - | - | - | - | 3.42% | 3.22% | - | 3.22% | 4.23% | 74.25% | 39.03% | 3.02% |
| **GI 1 (STF** ↑**)** |  |  |  |  |  |  |  |  |  |  |  |  |
| DC | 5.39% | 5.88% | 2.45% | - | 7.35% | 6.86% | 14.23% | 21.08% | 11.76% | 8.82% | 7.35% | 20.1% |
| ReHo | 3.03% | 5.23% | 0.55% | - | 4.68% | 20.39% | - | 24.52% | 15.43% | 6.34% | 2.2% | 1.38% |
| **GI 2 (STF** ↓**)** |  |  |  |  |  |  |  |  |  |  |  |  |
| DC | 33.33% | 38.46% | 21.79% | 12.82% | 1.28% | - | 64.1% | 1.28% | - | 7.69% | 7.69% | - |
| ReHo | 30.33% | 76.23% | 36.89% | 5.74% | 18.85% | - | 81.15% | 28.69% | - | 0.82% | 5.74% | 1.64% |
| VMHC | 18.18% | 12.32% | 18.48% | 8.21% | 8.5% | 6.74% | 12.9% | 4.12% | 4.99% | 6.45% | 9.38% | 7.62% |
| PCC-iFC | 75% | 22.06% | 76.47% | 2.94% | - | 2.94% | 88.24% | 7.35% | - | 4.41% | 1.47% | - |

C1 = Motor (Hand); C2 = Motor (Oral); C3 = Auditory; C4 = Higher Order Visual; C5 = Language; C6 = Oculomotor; C7 = Sensory Visceral; C8 = Inhibitory Control; C9 = Cognitive Flexibility; C10 = Theory of Mind; C11 = Emotion Recognition; C12 = Reward; DC = degree centrality; fALFF = fractional amplitude of low frequency fluctuations; PCC-iFC = posterior cingulate cortex intrinsic functional connectivity; ReHo = regional homogeneity; VMHC = voxel-mirrored homotopic connectivity; STM = shift-towards-maleness; STF = shift-towards-femaleness; turquoise: EMB 1 = ASD♂>NT♂ & NT♂>NT♀; blue: EMB 2 = ASD♂<NT♂ & NT♂<NT♀; orange: GI 1 = ASD♂>NT♂ & NT♂<NT♀; yellow: GI 2 = ASD♂<NT♂ & NT♂>NT♀.
