# Supplementary material for: Network-specific sex differentiation of intrinsic brain function in males with autism
Source: Mol Autism. 2018 Mar 6;9:17. doi: 10.1186/s13229-018-0192-x (PMC5840786; doi:10.1186/s13229-018-0192-x)
Supplement: Supplementary file 13 — Similarity across analytical strategies. (DOCX 61 kb) [file 13229_2018_192_MOESM13_ESM.docx]

**Additional File 13: Table S5. Similarity across analytical strategies**

| **Strategies** | **Sources of Differences** | **AO^a^ % Across Strategies** |
| --- | --- | --- |
| **Strategies 1 and 2** | Individual preprocessing & group level covariates | 16.81% |
| **Strategies 1 and 3** | Individual preprocessing, group level covariates & age | 9.87% |
| **Strategies 1 and 4** | Individual preprocessing, group level covariates, age & samples | 8.61% |
| **Strategies 2 and 3** | Age | 21.41% |
| **Strategies 2 and 4** | Samples & age | 13.93% |
| **Strategies 3 and 4** | NT Sample | 38.28% |

^a^AO = Average Overlap across all 500 voxel-level thresholds; Strategy 1 = original Z-maps; Strategy 2 = conjunction of Z-maps obtained with a common analytical pipeline in ABIDE I and FCP samples; Strategy 3 = analyses conducted with age-matched subsamples and a common pipeline; Strategy 4 = replication with an independent sex difference sample (GSP) using age-matched subsamples and a common pipeline.
