# Supplementary material for: Network-specific sex differentiation of intrinsic brain function in males with autism
Source: Mol Autism. 2018 Mar 6;9:17. doi: 10.1186/s13229-018-0192-x (PMC5840786; doi:10.1186/s13229-018-0192-x)
Supplement: Supplementary file 14 — Similarity across R-fMRI metrics by model across all analytical strategies. (DOCX 80 kb) [file 13229_2018_192_MOESM14_ESM.docx]

**Additional File 14: Table S6. Similarity across R-fMRI metrics by model across all analytical strategies**

|  | **All R-fMRI Metrics** | **DC** | **fALFF** | **ReHo** | **VMHC** | **PCC-iFC** |
| --- | --- | --- | --- | --- | --- | --- |
| **Models** | **AO %** | **AO %** | **AO %** | **AO %** | **AO %** | **AO %** |
| **EMB 1 (STM** ↑**)** | 10.00% | 4.55% | 10.43% | 25.71% | 4.76% | 4.57% |
| **EMB 2 (STM** ↓**)** | 30.43% | 8.26% | 18.99% | 36.31% | 43.87% | 44.72% |
| **GI 1 (STF** ↑**)** | 15.10% | 17.39% | 11.35% | 28.40% | 10.05% | 8.33% |
| **GI 2 (STF** ↓**)** | 17.07% | 14.78% | 22.39% | 16.03% | 20.96% | 11.18% |

AO = Average of percentage of overlap across all 500 voxel-level thresholds. STM = shift-towards-maleness; STF = shift-towards femaleness; turquoise: EMB 1 = ASD♂>NT♂ & NT♂>NT♀; blue: EMB 2 = ASD♂<NT♂ & NT♂<NT♀; orange: GI 1 = ASD♂>NT♂ & NT♂<NT♀; yellow: GI 2 = ASD♂<NT♂ & NT♂>NT♀.
